# Transcripts and MicroRNAs Responding to Salt Stress in *Musa acuminata* Colla (AAA Group) cv. Berangan Roots

**DOI:** 10.1371/journal.pone.0127526

**Published:** 2015-05-20

**Authors:** Wan Sin Lee, Ranganath Gudimella, Gwo Rong Wong, Martti Tapani Tammi, Norzulaani Khalid, Jennifer Ann Harikrishna

**Affiliations:** 1 Institute of Biological Sciences, Faculty of Science, University of Malaya, Kuala Lumpur, Malaysia; 2 Centre for Research in Biotechnology for Agriculture, University of Malaya, Kuala Lumpur, Malaysia; 3 Bioinformatics, Sime Darby Technology Centre Sdn Bhd, Serdang, Selangor, Malaysia; Northeast Forestry University, CHINA

## Abstract

Physiological responses to stress are controlled by expression of a large number of genes, many of which are regulated by microRNAs. Since most banana cultivars are salt-sensitive, improved understanding of genetic regulation of salt induced stress responses in banana can support future crop management and improvement in the face of increasing soil salinity related to irrigation and climate change. In this study we focused on determining miRNA and their targets that respond to NaCl exposure and used transcriptome sequencing of RNA and small RNA from control and NaCl-treated banana roots to assemble a cultivar-specific reference transcriptome and identify orthologous and *Musa*-specific miRNA responding to salinity. We observed that, banana roots responded to salinity stress with changes in expression for a large number of genes (9.5% of 31,390 expressed unigenes) and reduction in levels of many miRNA, including several novel miRNA and banana-specific miRNA-target pairs. Banana roots expressed a unique set of orthologous and *Musa*-specific miRNAs of which 59 respond to salt stress in a dose-dependent manner. Gene expression patterns of miRNA compared with those of their predicted mRNA targets indicated that a majority of the differentially expressed miRNAs were down-regulated in response to increased salinity, allowing increased expression of targets involved in diverse biological processes including stress signaling, stress defence, transport, cellular homeostasis, metabolism and other stress-related functions. This study may contribute to the understanding of gene regulation and abiotic stress response of roots and the high-throughput sequencing data sets generated may serve as important resources related to salt tolerance traits for functional genomic studies and genetic improvement in banana.

## Introduction

Crops worldwide are threatened by increased soil salinity due to accumulation of salt delivered along with irrigation water and by coastal flooding and the high evapotranspiration rates caused by climate change. Soil salinity has been predicted to increase to about 30% of the world’s arable land by 2025 and 50% by the year 2050 [1] and currently over 11% of irrigated areas of arable land have been salinized by irrigation [2]. One promising approach to address the problem of soil salinity is to increase our understanding of response of plants to salinity-related stress. Yield of cultivated banana (the world’s most important fruit crop) has been found to be reduced by about 50% [3] and plant height was reduced by about 75% [4] under salt stress. Since banana is sensitive to salt stress, it represents a model for investigating the genetic sources of salt sensitivity.

Plant stress response mechanisms have been broadly categorised into stress avoidance, and stress tolerance, the latter being used when stress is severe [5]. Increased levels of salinity stress affect plants by a combination of acute osmotic stress, followed by Na^+^ and Cl^-^ ion toxicity [6]. A common avoidance response to high salinity is a change in root growth, or negative halotropism [7] with the response varying in response to the gradient, timing and duration of stress (reviewed in [5,6]). Although several stress response genes and pathways are starting to be understood, including signal transduction, activation of ion channels and growth factor regulated modification of plant architecture and in particular, root morphology, the complexity and interaction of the pathways, and differences between plants, leave much to be learned about the genetic regulation of plant responses and for the development and selection of salinity stress tolerant crops.

MicroRNAs (miRNA) regulate diverse life processes in plants, largely through the negative regulation of transcription factors, and have important roles in growth, development, physiology, stress responses and RNA interference pathways including miRNA and siRNA biogenesis (reviewed in [8]). A number of miRNA families have been shown to modulate various abiotic stress responses in plants, including responses to salinity (reviewed in [9]). Patterns of miRNA expression in various plant tissues and under various salinity conditions are being revealed through high-throughput based studies, for example in *Zea mays* root [10], *Arabidopsis thaliana* seedlings [11], *Populus euphratica* shoots [12], *Saccharum sp*. leaves [13], *Triticum aestivum* L. seedling leaves [14], *Brassica juncea* (Czern) L. seedlings [15], switchgrass seedlings [16] and *Gossypium hirsutum* L. leaves and roots [17]. However, relatively few studies have focused specifically on roots, despite this being the main tissue exposed and reacting to salinity.

In this study we used a deep sequencing approach to investigate the expression and role of miRNA in banana roots in order to identify orthologous and new miRNAs related to response to salinity. We also predict targets of the differentially expressed miRNAs which may have roles in the response of banana roots to salinity stress.

## Results

### Transcriptome

#### *De novo* assembly of transcriptomes and mapping of assembled sequences to the *Musa acuminata* reference genome (A-genome)

**Fig 1:**
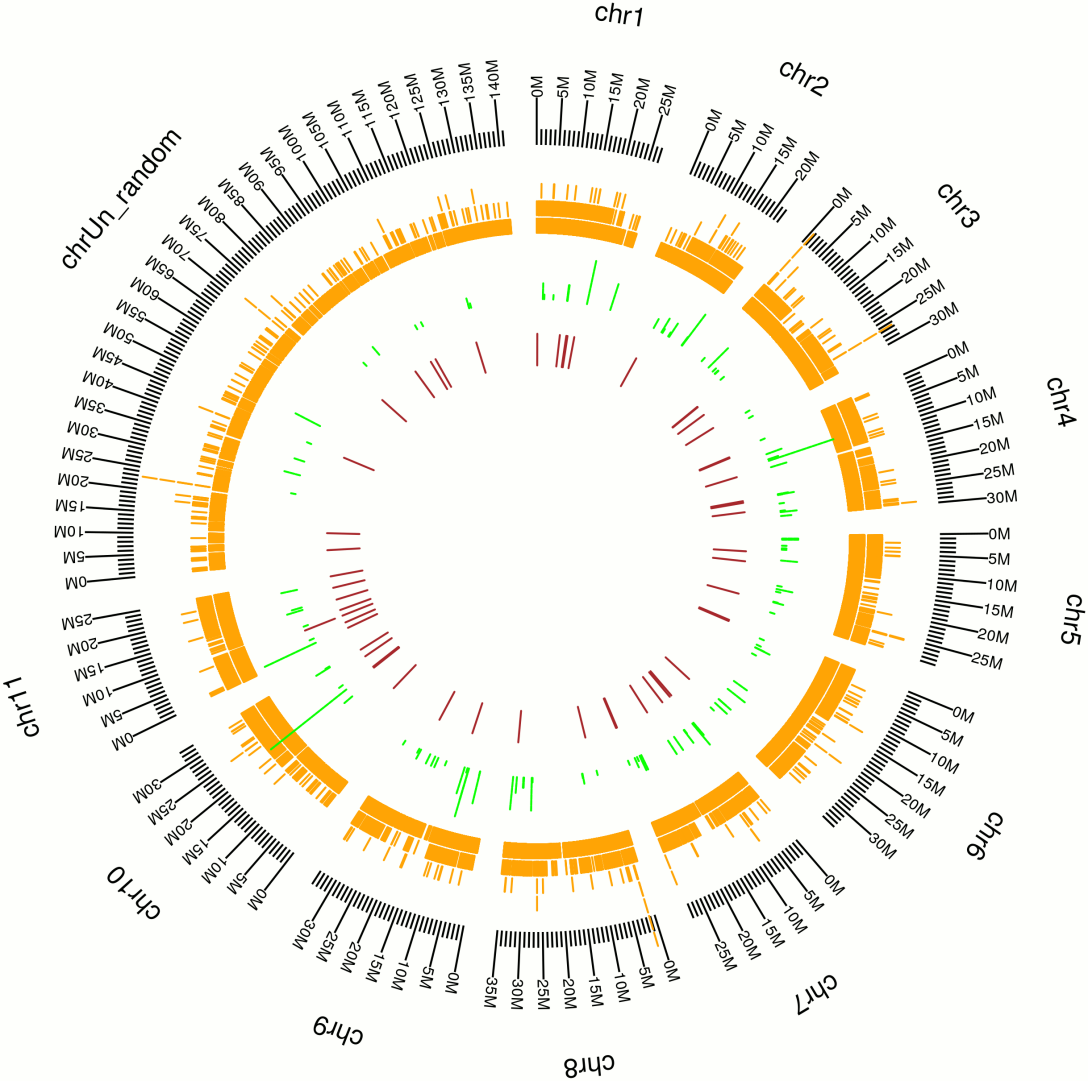
Distribution of expressed transcripts on a reference *Musa* Genome [18]. Black (outer ring): chromosomes of the reference *Musa* A-genome, scale in Mb; orange: *de novo* assembled unigenes; green: orthologous microRNAs; maroon: *Musa*-specific microRNAs.

Over 12.2 million and 11.2 million, 90-bp paired-end reads were generated from non-stressed control and 300 mM NaCl treated roots, respectively (Table A in S1 File). These paired-end sequences in FASTQ format were deposited in Short Read Archive (SRA) of National Centre for Biotechnology Information (NCBI) and are available under BioProject accession PRJNA246442. All together these reads accounted for 2.1 Gb of transcriptomic sequence data. Both transcriptomes contained more than 92% of high quality reads and more than 93% of high quality bases (Phred score Q>20) (Table A in S1 File). The 11,352,903 and 10,504,837 high quality reads (Table A in S1 File) from each data set were assembled into 69,441 and 74,525 contigs representing 49,576 and 56,572 unigenes from the control and NaCl-treated roots, respectively and were used to form a set of non-redundant representative transcripts containing 31,390 unigenes with a mean length of 517-nt and an N50 of 669-nt (Table B in S1 File). The mean coverage per base (CPB) per unigene of the assembled transcriptomes is 45.33X in CTR and 38.57X in TR300 (Table C in S1 File). More than 80% of the contigs assembled in CTR and TR300 were sized 100–400 nt (Figure A in S1 File). The assembled sequences showed increased length after assembly and clustering into unigenes (Figure A in S1 File). More than 80% of the assembled scaffolds and unigenes are of high quality with zero ambiguous nucleotides (Figure B in S1 File). Over 99.5% of our *de novo* assembled unigenes can be mapped to the *Musa* genome [18] (Fig 1 and Table D in S1 File). These unigenes were distributed along the 11 chromosomes and unrandom sequences of the *Musa* reference genome. The number of unigenes that could be mapped to each chromosome ranged from 2,000 to 3,200, or 6 to 10% of the unigenes (Table D in S1 File).

#### Functional assignment of the banana transcriptomic sequences

Of the 31,390 clustered unigenes, 70.8% (22,231) found matches in the GenBank non-redundant protein database (Nr) and 47.5% (14,913) in the UniProt (Table 1). A majority of the BLAST hits are *Oryza sativa* protein sequences in the Nr database, followed by *Vitis vinifera*, *Glycine max*, *Populus trichocarpa*, *Arabidopsis thaliana* and *Zea mays* (Figure C in S1 File). About 56% or 17,617 of the assembled unigenes were assigned to at least one Gene Ontology (GO) term, 30% or 9,449 to 25 categories in Eukaryotic Orthologous Groups of Proteins (KOG), and 22.2% or 6,997 to 136 pathways in Kyoto Encyclopedia of Genes and Genomes (KEGG) (Table 1).

**Table 1:** Functional annotation of banana root transcriptome (all unigenes).

	Number of unigene	Percentage (%)
**Total**	31,390	100
**Nr**	22,231	70.8%
**UniProt**	14,913	47.5%
**GO**	17,617	56.1%
**KEGG**	6,997	22.2%
**KOG**	9,449	30%

Nr: GenBank non-redundant protein database; GO: Gene Ontology; KEGG: Kyoto Encyclopaedia of Genes and Genomes; KOG: Eukaryotic Orthologous Groups of Protein.

#### Transcripts quantification and differential expression in salt-stressed banana roots

**Fig 2:**
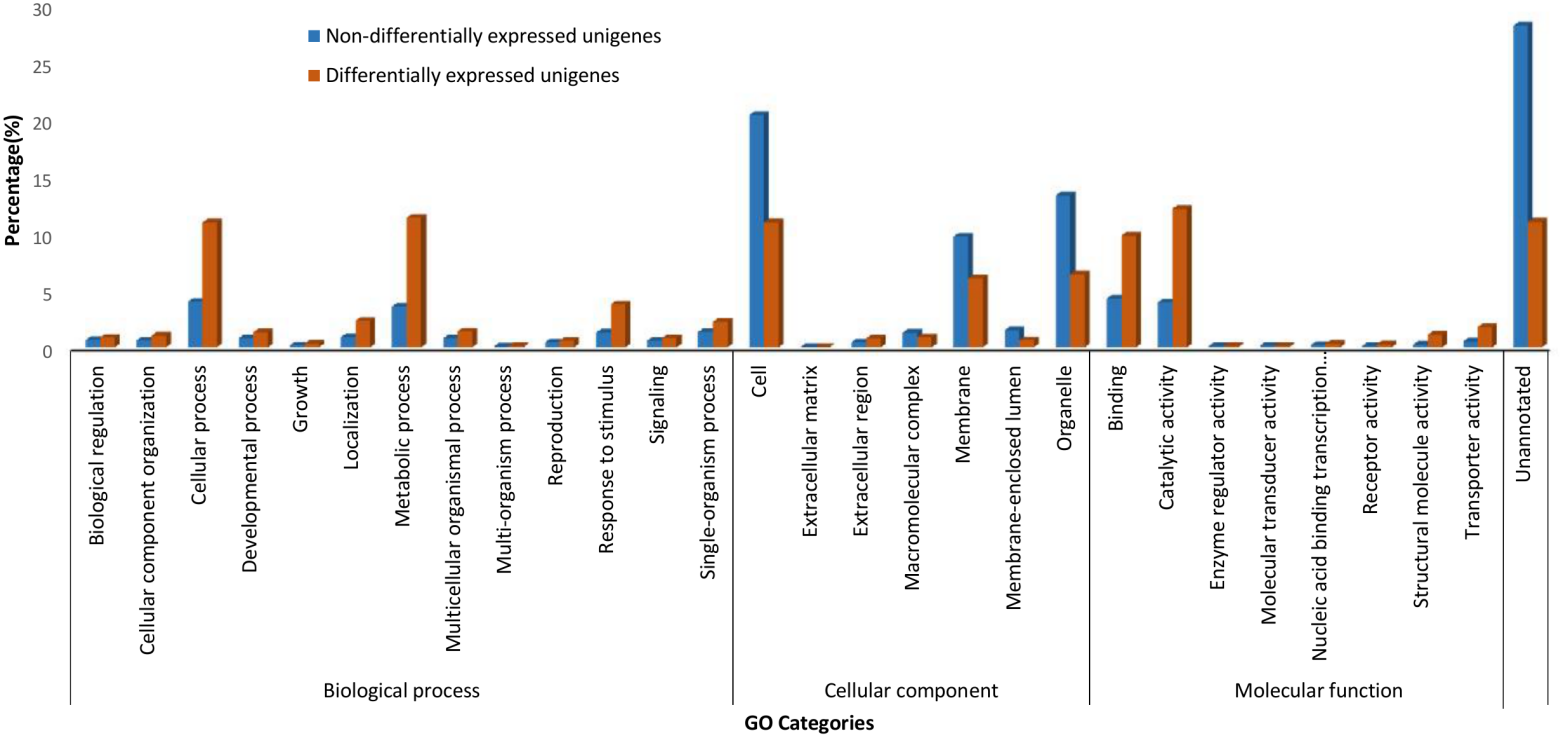
Gene Ontology (GO) assignments. GO assignment for the transcripts (unigene) differentially-expressed and non-differentially-expressed in the salt-stressed banana roots.

After being normalized to Transcript Per Million (TPM), 9.5% or 2,993 of the *de novo* assembled unigenes were observed to be differently expressed in salt-stressed banana root with absolute log_2_(CTR/TR300) equal to or higher than 1 and False Discovery Rate < 0.05 (Fig 2). More transcripts (unigenes) were up-regulated (57.5% or 1,720) than down-regulated (42.5% or 1,273) in banana roots upon 300 mM NaCl treatment (Fig 2). Among these unigenes, the largest proportion was assigned to the catalytic activity (molecular function) and metabolic process (biological process) enriched Gene Ontology terms (Fig 2). A majority of the differentially-expressed unigenes in the salt-stressed banana roots were assigned to carbohydrate transport and metabolism, general function prediction, and post-translational modifications, protein turnover, chaperones of the Eukaryotic Orthologous Groups of Proteins (KOG) (Figure D in S1 File). In KEGG pathways assignment, the differentially expressed unigenes were predominantly classified in the starch and sucrose metabolism pathways, followed by phenylalanine metabolism, cysteine and methionine metabolism, and phenylpropanoid biosynthesis pathways (Figure E in S1 File).

### microRNA

#### Overview of the small RNA libraries

Deep sequencing of small RNA libraries constructed from banana roots treated with low salinity (100 mM NaCl), TR100, high salinity (300 mM NaCl), TR300 and untreated control, CTR using an Illumina GAxII sequencing platform generated more than 18 million tags in each library. About 14.8, 13.8 and 14.5 million high quality clean reads ranging from 17–30 nt were produced respectively from TR100, TR300 and CTR and used for small RNA (miRNA) annotation (Table E in S1 File). The Illumina small RNA sequence reads described in this study are available under NCBI’s BioProject accession PRJNA246442. About 1.91 (CTR), 1.75 (TR100) and 0.91 (TR300) million non-redundant sequence reads (~53% to 60% of the non-redundant reads), could be mapped to the reference *Musa* genome [18] (Table E in S1 File). The 21-nt sequence reads were dominant in all the three libraries, with 20-nt or 24-nt reads the next most abundant. Unlike CTR and TR100, TR300 had a higher percentage of 20-nt sRNA than 24-nt (Figure F in S1 File). Rfam and Plant MicroRNA Database (PMRD) annotation of the small RNA sequences (Figure G in S1 File) showed that the vast majority (>88%) of the sequence reads in the banana root sRNA libraries are sequences with no known function. Among the reads annotated with an identity, miRNA sequences were the most abundant forming 6–10% of the sRNA libraries (Figure G in S1 File). The second most abundant small RNA with an identity was rRNA, forming 1–2% in each library while each of the other known small RNAs (tRNA, snRNA and snoRNA) formed less than 1% of the sRNA libraries (Figure G in S1 File).

#### Annotation of miRNAs and their distribution in the A-genome

Matching our small RNA dataset to the Plant MicroRNA Database (PMRD- http://bioinformatics.cau.edu.cn/PMRD/), we identified 153 (39 families), 149 (40 families) and 128 (35 families) orthologous miRNA sequences in CTR, TR100 and TR300 small RNA libraries respectively (Table F in S1 File). Using our RNA-Seq transcriptome data, we identified 110, 115 and 91 target RNA transcripts for the orthologous miRNAs present in CTR, TR100 and TR300 respectively (Table F in S1 File). All together, 181 miRNAs from 47 miRNA families, and a non-redundant set of 247 related target transcripts were found in this study (Table F in S1 File). From the deeply sequenced banana root sRNAomes in this study, we identified 56 putative *Musa*-specific miRNAs (non-redundant), which are not reported in species other than *Musa* species to date, and 120 predicted target transcripts (Table G in S1 File). When mapped to the genome sequence, both the orthologous and *Musa*-specific miRNAs were distributed in all 11 chromosomes and the unrandom sequence of the reference banana genome as shown in Fig 1.

#### Differentially-expressed miRNAs in the 100 mM and 300 mM NaCl-treated banana roots

**Fig 3:**
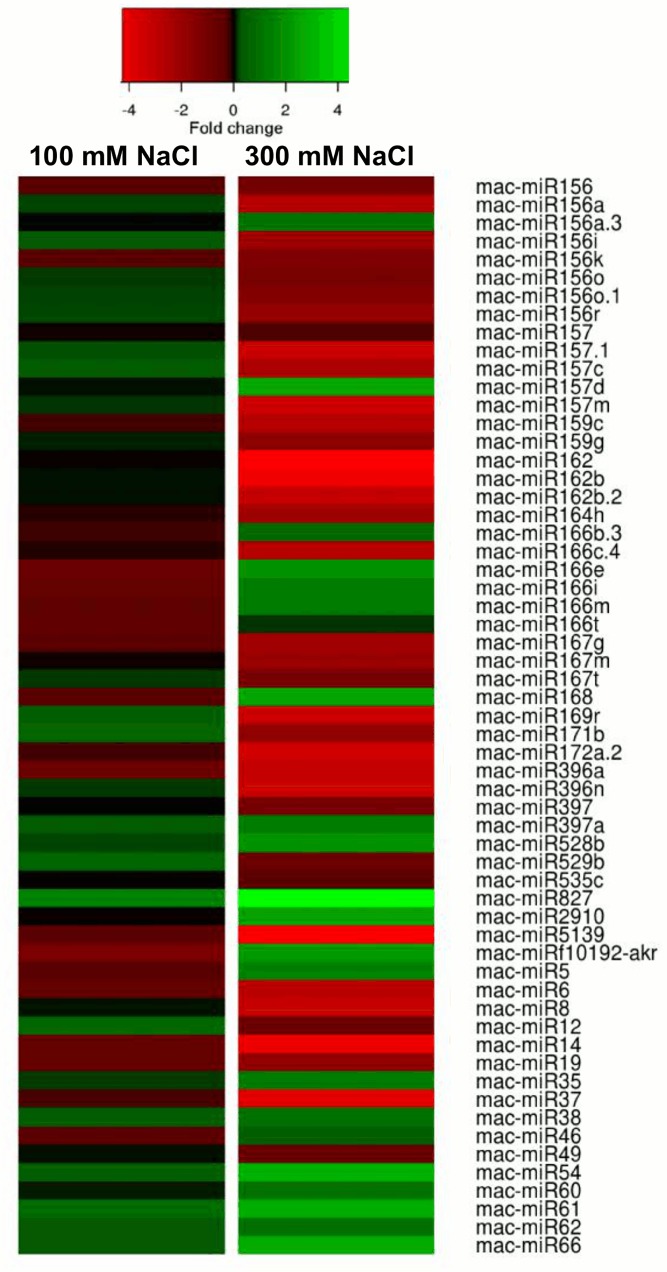
Orthologous and *Musa*-specific miRNAs differentially expressed in 100 mM and 300 mM NaCl. Statistically significant (absolute log_2_ fold change ≥ 1 and FDR cut-off < 0.05) changes in miRNA expression in banana roots treated with 100 mM and 300 mM in comparison to untreated control (0 mM NaCl).

We found 43 orthologous miRNAs (belonging to 20 miRNA families) and 16 *Musa*-specific putative miRNAs were differentially expressed upon salt stress in banana roots in either 100 mM or 300 mM NaCl treatment, in comparison to the control (Fig 3). These miRNAs showed different expression patterns when different magnitudes of salt stress (100 mM and 300 mM NaCl) were applied (Fig 3).

Some of these miRNAs showed increased expression levels in both 100 mM and 300 mM NaCl. These include mac-miR397a, mac-miR528, mac-miR827, mac-miR35, mac-miR38, mac-miR54, mac-miR60, mac-miR61, mac-miR62 and mac-miR66 (Fig 3). Whereas miRNAs including mac-miR156, mac-miR156k, mac-miR159c, mac-miR164h, mac-miR166c.4, mac-miR167g, mac-miR172a.2, mac-miR396a, mac-miR5139, mac-miR8, mac-miR14, mac-miR19 and mac-miR37 were among the miRNAs down-regulated in both 100 mM and 300 mM NaCl (Fig 3).

Some of these differentially expressed miRNAs were up-regulated in 100 mM NaCl but down-regulated in 300 mM. These include mac-miR156a, mac-miR56i, mac-miR156o, mac-miR156o.1, mac-miR156r, mac-miR157m, mac-miR159g, mac-miR162b, mac-miR162b.2, mac-miR167t, mac-miR169r, mac-miR171b, mac-miR396n, mac-miR529b, mac-miR12 and mac-miR49 (Fig 3). Whereas miRNAs, including mac-miR166b.3, mac-miR166e, mac-miR166i, mac-miR166m, mac-miR166t, mac-miR168, mac-miRf10192-akr, mac-miR5, mac-miR46 and mac-miR49, were down-regulated in 100 mM NaCl but up-regulated in 300 mM NaCl (Fig 3). Some of the miRNAs showed other expression patterns, such as up-regulation (mac-miR156a.3 and mac-miR2910) or down-regulation (mac-miR162, mac-miR167m, mac-miR397 and mac-miR535) in TR300 but no change in expression in TR100.

A majority of the targets (found in the assembled unigenes) for these differentially expressed miRNAs are functionally related to binding (molecular function), cellular process (biological process), cell (cellular component) and membrane (cellular component) as shown in Figure H in S1 File.

#### Differentially-expressed miRNAs in 300 mM NaCl-treated banana roots and their predicted targets

**Fig 4:**
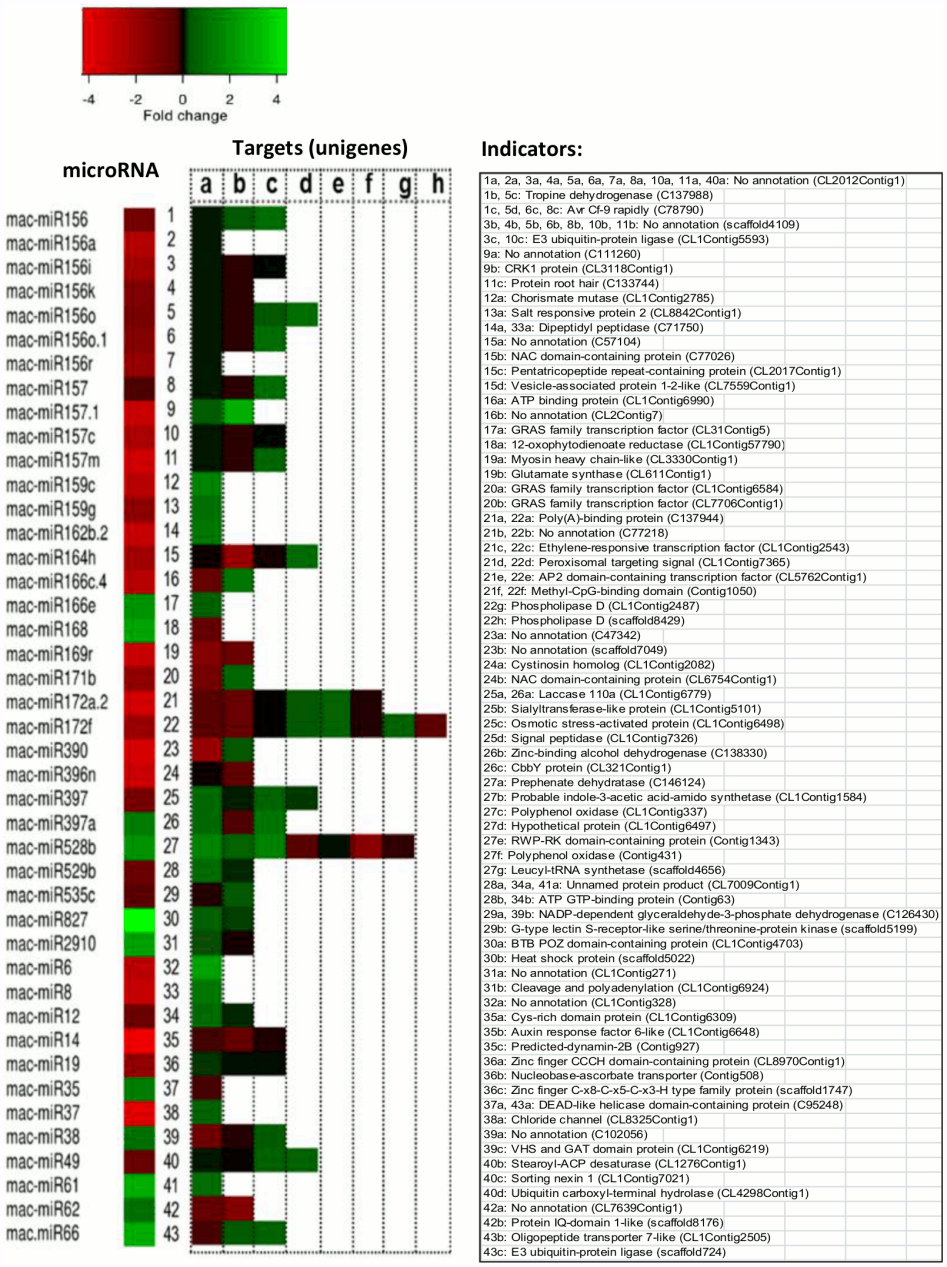
Differentially-expressed miRNAs in 300 mM NaCl. Orthologous and *Musa*-specific miRNAs with statistically significant expression change (absolute log_2_ fold change ≥ 1 and FDR cut-off < 0.05) in banana roots upon 300 mM NaCl treatment, and their corresponding targets (unigenes) in the RNA-Seq data. Numbers and letters refer to the predicted target sequences as shown in the right panel.

Forty three miRNAs (both orthologous and *Musa*-specific) were differentially expressed in 300 mM with at least one target in the banana root transcriptome (Fig 4). Most of these differentially expressed miRNAs showed an inverse expression pattern to at least one of their predicted targets, with the exception of mac-miR166e, mac-miR169r, mac-miR396n, mac-miR827, mac-miR14 and mac-miR61 (Fig 4).

We found 12 *Musa*-specific miRNAs, which have not been reported yet in plants other than *Musa* species, differentially expressed in salt-stressed banana roots (300 mM NaCl) with at least one target found in the banana root transcriptomes (Fig 4). Among the 12 *Musa*-specific miRNAs that were differentially expressed, sequences of two miRNAs (mac-miR6 and mac-miR19) were previously reported in *Musa balbisiana* [19].

A majority of the differentially-expressed miRNAs were down-regulated (Figs 3 and 4) while at least one of their predicted targets were up-regulated in the salt-stressed banana roots (TR300). These include mac-miR156, mac-miR156a,i,k,o,o.1,r, mac-miR157, mac-miR157.1, mac-miR157c,m, mac-miR159c,g, mac-miR162b.2, mac-miR164h, mac-miR166c.4, mac-miR171b, mac-miR172a.2, mac-miR172f, mac-miR390, mac-miR397, mac-miR529b, mac-miR535c, mac-miR6, mac-miR8, mac-miR12, mac-miR19, mac-miR37 and mac-miR49 (Fig 4).

Some differentially-expressed miRNAs were up-regulated (including mac-miR168, mac-miR397a, mac-miR528b, mac-miR35, mac-miR38, mac-miR62 and mac-miR66) and at least one of their targets were down-regulated (Fig 4). A small number of the differentially-expressed miRNAs showed the same expression pattern (either up- or down-regulation) as their targets, as shown in Fig 4. These miRNAs include mac-miR166e, mac-miR169r, mac-miR396n, mac-miR827, mac-miR2910, mac-miR14 and mac-miR61.

Among the predicted targets of the differentially expressed miRNAs are transcription factors, including AP2 domain-containing transcription factor (targeted by mac-miR172), GRAS family transcription factor (targeted by mac-miR166 and mac-miR171) and ethylene-responsive transcription factor (targeted by mac-miR172); transcripts coding for stress responsive proteins, such as, salt responsive protein (targetted by mac-miR159), osmotic stress-activated protein (targetted by mac-miR397), heat shock protein (targetted by mac-miR827); transcript coding for structural proteins such as protein root hair (targetted by mac-miR157), chloride channel (targetted by mac-miR37) and oligopeptide transporter 7-like (targetted by mac-miR66); enzymes involved in metabolism, such as, glutamate synthase (targetted by mac-miR169) and tropine dehydrogenase (targetted by mac-miR156), as well as transcripts coding for unknown or hypothetical proteins (Fig 4).

Among the unknown transcripts predicted to be targetted by the differentially-expressed miRNAs, only one transcript, unigene CL1Contig328, was found with a domain annotation using InterProScan (http://www.ebi.ac.uk/Tools/pfa/iprscan/). This unigene, which is targetted by mac-miR6, contains a dehydrin domain (InterProScan ID: IPR000167).

#### Experimental validation of salinity-stress induced miRNA and mRNA targets

**Fig 5:**
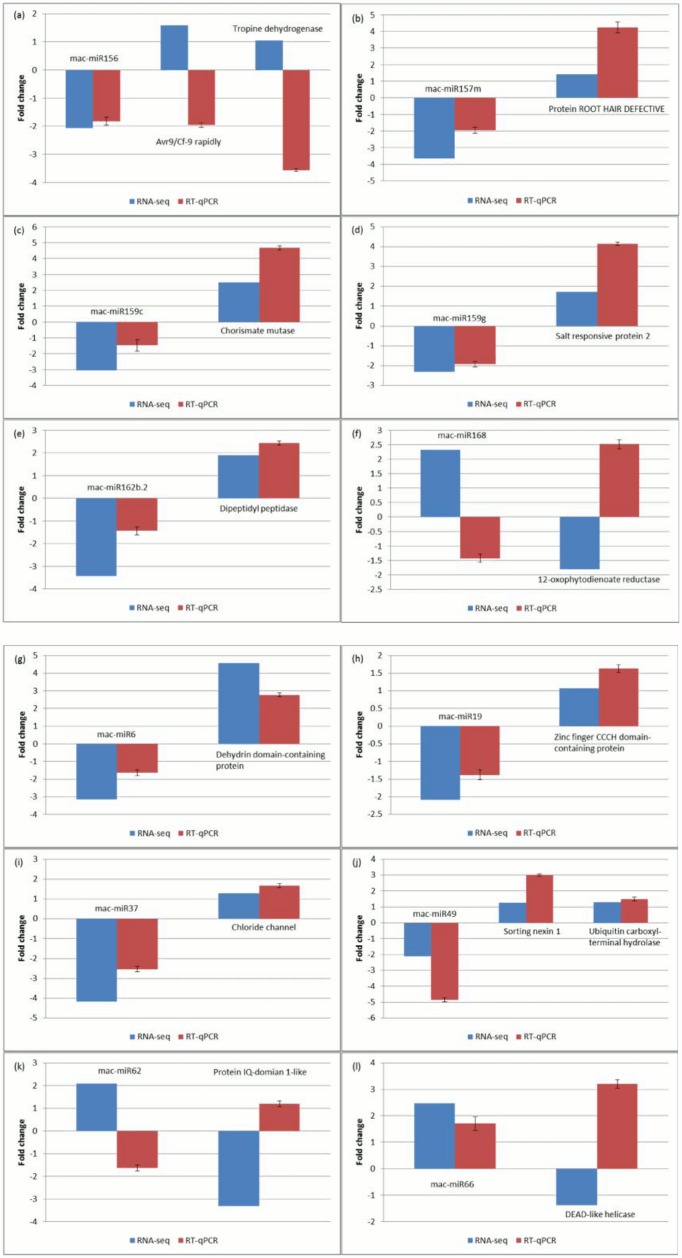
Real-time quantitative PCR analyses of selected miRNAs and their predicted target mRNAs in root tissues of banana plantlets grown under 300 mM NaCl treatment. MiRNA and their corresponding target mRNA are arranged to appear together in each graph (a)-(f) plant orthologous miRNA; (g)-(l) *Musa*-specific miRNA. Fold changes in gene expression shown are relative to the expression in the non-stressed roots (control). RNA-seq and PCR data are both shown in linear scale. Errors bars indicate mean ± standard deviations obtained from three biological replicates.

We performed RT-qPCR for 12 of the miRNAs that were determined as differentially expressed in salt-stressed banana root transcriptomes and for 14 of their corresponding targets, in order to validate the RNA-seq data. The miRNA were selected to include 6 plant orthologous miRNA and 6 *Musa*-specific miRNA. The RT-qPCR results showed general agreement with the gene expression patterns determined by RNA-seq (Fig 5b, 5c, 5d, 5e, 5g, 5h, 5i and 5j), with only 2 out of the 12 miRNA and 5 of the 14 miRNA-target profiles showing discrepancy between the methods (Fig 5a, 5f, 5k and 5l).

## Discussion

Salinity adversely affects productivity and quality of crops and the world-wide increase in salinization of arable land poses a risk to global food security. Cultivated bananas are salt sensitive and so an understanding of the molecular basis of their salt stress response may help when forming strategies for improving salinity tolerance in banana and other economically important crops. Plant roots are the primary site of perception for water-limiting stress, including salinity and drought [20]. Thus understanding the genetic regulation underlying the physiological changes in roots in response to salinity stress, are key to the development of strategies for crop improvement in the face of environmental degradation and climate change. In this study we applied high throughput sequencing to profile transcriptomes, including the functional small RNA, of salt-stressed banana roots.

### High quality *de novo* salt stress transcriptome assembly for *M*. *acuminata* cv. Berangan

A large number of banana varieties are grown around the world, and while most cultivars are comprised of either A or A and B genomes, which both have published reference genome sequences [18,19], transcriptome data adds useful information on gene expression in addition to providing cultivar-specific data for better prediction of miRNA targets. Here, around 3 Gbp of gene expression data from banana roots (two transcriptomes of ~2.1 Gbp and three small RNA libraries of ~ 900 Mbp) provided data for the assembly of 31,390 unigenes, which compares well with the 36,542 and 36,638 predicted protein-coding gene models in the reference genomes for *Musa acuminata* [18] and *Musa balbisiana* [19] respectively. The unigenes assembled from Illumina HiSeq 2000 data have a mean length of 517 bp, which is only 6.7% shorter than the previously reported banana root transcriptome assembled from 9.3 Gbp data generated using the same sequencing platform [21]. More than 99.5% of the unigenes from the *de novo* transcriptome assembly in this study can be mapped to the A-genome with a high degree of matching (mis-match score of <3) reflecting the high quality of the assembly.

### Salt stress-responsive genes in banana roots

A sizable proportion of the unigenes (9.5%) and miRNA (~25%) showed differential expression between salinity stress and control conditions, suggesting that a large number of genes and miRNA are involved in the banana salinity response. The functional roles of annotated genes that were differentially regulated in response to elevated salinity, were broadly similar to those reported for other plants. Catalytic activities, metabolic processes, cellular processes, binding, and cell and cell parts are the highly represented GO terms observed in this present study (Fig 2), and these GO terms are generally enriched in response to salt stress in other plants, for example, in salt-stressed roots in soybean [22], in two desert poplar species [23], in the leaf tissues of *Petunia hybrida* [24] and in a recretohalophyte, *Reaumuria trigyna* [25]. Enrichment in these GO terms may indicate plant plasticity in response to salt stress through switches of biochemical and morphological activities in cells.

The KEGG pathways enriched in this present study, were predominantly classified in the starch and sucrose metabolism, phenylalanine metabolism, cysteine and methionine metabolism, and phenylpropanoid biosynthesis pathways (Figure E in S1 File), as also observed in transcriptomes of other plants, such as those of rice roots, in response to salt stress [26]. Sugar metabolism and mobilization are important for osmotic adjustment and stress adaptation in plants [27, 28]. It has been reported that salt tolerance is correlated with metabolism and allocation of carbohydrate in the leaves, stems and roots where hexoses accumulated and induced a feedback repression to photosynthesis in the salt-stressed perennial ryegrass [29]. Phenylalanine, cysteine and methionine are important amino acids in plants. Phenylalanine serves as a precursor for the synthesis of various vital metabolites in plants [30], whereas cysteine is a precursor for the biosynthesis of various important molecules, including vitamins, cofactors, antioxidants and compounds for defence [31]. Methionine is an important precursor or donor for the metabolism of various other primary and secondary metabolites [32]. The regulation of genes involved in these metabolic pathways may help banana roots to cope with salt stress through the production of a broad range of metabolites, as has previously been reported in various plants (reviewed in [33]). In addition, the deep-sequencing approach used in our study revealed a large number of unannotated genes found to be differentially expressed in banana roots during salt stress (Fig 2). Future investigation to study their functions, may elucidate additional mechanisms for salinity stress tolerance with potential for application in combating abiotic stress in crops such as banana. Interestingly, some of the unannotated genes are predicted targets of salinity stress-responsive miRNA, including the dehydrin-domain carrying CL1Contig328, a predicted target of novel Musa-specific mac-miR6, whose inverse expression patterns were confirmed by RT-qPCR in the current study (Fig 5g).

**Fig 6:**
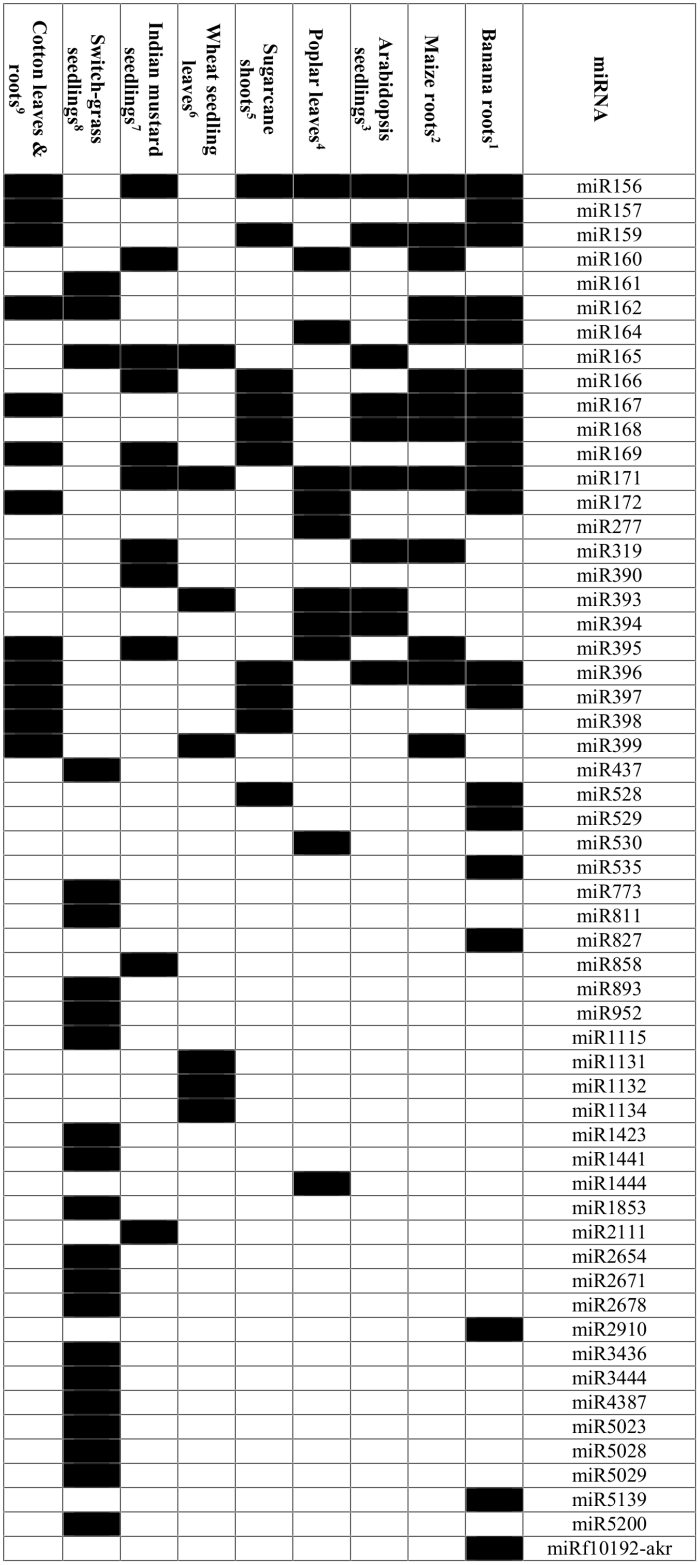
Salt-stress responsive miRNAs. Salt-stress responsive miRNAs are the statistically significant differentially-expressed miRNAs reported in the following studies: 1. Present study with *Musa acuminata* roots; 300 mM NaCl treatment; sRNA-Seq; log_2_ fold change ≥1 or ≤-1 and FDR < 0.05; 2. *Zea mays* roots; 200 mM NaCl treatment; miRNA microarray; P-value < 0.01 [10]; 3. *Arabidopsis thaliana* seedlings; 300 mM NaCl treatment; miRNA microarray; fold change > 1.5 and q value < 0.001 [11]; 4. *Populus euphratica* shoots; 200 mM NaCl treatment; sRNA-Seq; miRNAs with reverse expression with their targets [12]; 5. *Saccharum* sp. leaves; 170 and 340 mM NaCl treatments; sRNA-Seq; validated by RT-qPCR, with p<0.05 [13]; 6. *Triticum aestivum* L. seedling leaves; 300 mM NaCl treatment; RT-qPCR; fold change > 1.5 [14]; 7. *Brassica juncea* (Czern) L. seedlings; 150 and 250 mM NaCl treatments; sRNA-Seq; log_2_ fold change ≥1 or ≤-1 [15]; 8. *Panicum virgatum* seedlings; 0.5% NaCl; sRNA-Seq; log_2_ fold change ≥1 or ≤-1 and P-value ≤0.01 [16]; 9. *Gossypium hirsutum* leaves and roots; 0.1, 0.25 and 0.5% NaCl treatments; RT-qPCR [17]

The high proportion of miRNA responding to stress seen in our data, is also observed in data from similar studies [14,15] and probably reflects the high level of regulatory changes required to achieve physiological responses to extreme conditions. Functional annotation of the differentially expressed genes, reflected the changes expected in severely stressed tissues, involving signaling, transport, defense and repair pathways, similar to the observations of changes in the banana proteome in response to drought [34]. Here, we chose to focus on the transcripts (mRNA) that were predicted targets of differentially expressed miRNA with an aim to use the changes in expression observed in the complementary data sets to validate one another. Among the orthologous miRNA sequences that were differentially expressed in response to salinity treatments, many have previously been reported from similar genomic-based studies in other plants (Fig 6), validating the quality of the small RNA data in the current study.

### Novel miRNA and miRNA-functions revealed by small RNA high-throughput sequencing of banana roots

Several orthologous miRNA were represented in one or more of the experimental conditions (i.e. roots in control, 100mM NaCl or 300mM NaCl). While the predicted targets for many of these were as reported for other plants, some were predicted to have targets not previously associated with those miRNA families (Table F and Table H in S1 File). Among the functions of the novel targets of orthologous miRNA were stress signaling, transcriptional and translational regulation, transport and cellular homeostasis, stress defence and metabolism. Additionally, 56 novel *Musa*-specific miRNA were predicted together with several novel mRNA targets from the assembled transcriptome data (Tables G and H in S1 File). Among these, are a few stress-related mRNAs that have been reported to be a target of a known miRNA, such as AUXIN RESPONSE FACTOR 6 (predicted target of mac-miR14 and miR167) and DEAD-box helicases (predicted target of mac-miR35, mac-miR66, miR164 and miR408) and thus may be under the regulation of more than one miRNA, or a different miRNA in banana (Fig 4). While there are many reports of orthologous miRNA with conserved functions and targets, including those responding to salinity stress, differences between the miRNA families reported as responding to salinity in different plants (Fig 6) may also reflect differences between the treatments and the tissues sampled. For example it has been observed that gene expression patterns change at different times after exposure to salinity stress and that differences in nutrient availability affect salt sensitivity in the root (reviewed in [5]).

### MiRNAs responded to salt stress in a dose-dependent manner

The varying response of the miRNA to the different levels of NaCl applied to banana roots (Fig 3) suggests the possibility of different sets of miRNA functions, depending on the magnitude of stress. This would be expected, as physiological responses to drought stress, for example, depend on stress severity, as well as on duration and the stage of plant life cycle [35]. Thus the overall genetic regulation, including the set of miRNA induced or repressed, is likely to vary with the environmental conditions to which the plant is exposed.

### MicroRNAs were predicted to target important salt stress-responsive genes in banana roots

The number of miRNAs that are differentially expressed following exposure to high salinity in banana roots, leads us to suggest that a large network of miRNA and miRNA-regulated genes may be directly involved in plant adaptation and survival. Most of the differentially-expressed miRNAs showed an inverse expression pattern with at least one of their predicted targets (Fig 4), as would be expected if mRNA cleavage occurs. This was also validated by RT-qPCR for most (8/12) of the miRNA-mRNA target pairs tested (Fig 5). RNA cleavage is the major mode of posttranscriptional gene silencing via miRNAs in plants, although there are several cases where miRNA targets are translationally inhibited but not cleaved (reviewed in [8]), and this may be the case for the differentially expressed miRNAs not showing an inverse expression pattern with their predicted target mRNA. However, it is possible that some of the predicted targets are not true targets of the miRNAs in banana root and additional functional confirmation would be required to rule this out. In our discussion here, we focus on miRNA and targets showing inverse expression profiles under the same conditions, with an aim to improve confidence in the predictions from the transcriptome and small RNA data. We also note that differential expression for 5/6 plant orthologous miRNA and 5/6 *Musa*-specific miRNA under salinity stress could be validated when tested in RT-qPCR (Fig 5).

Several of the predicted miRNA-target pairs that were differentially expressed during salinity stress in banana roots, have functions relating to stress response, adaptation and survival in plants. Of particular interest are those related to root development, which included miRNA families showing reduced expression with corresponding targets having increased expression levels in salinity stressed roots: Four distinct miR157 sequences were down-regulated in the salt-stressed banana roots, while their predicted target mRNAs including CDPK-related kinase (CRK1) and ROOT HAIR DEFECTIVE 3 (RHD3) were up-regulated (Figs 4 and 5b). Transcriptome data (RNA-seq) studies in Arabidopsis roots showed CDPK-related kinase to be induced in response to phosphate deprivation and have an association with root hair development [36]. RNA-seq of white lupin roots showed that ROOT HAIR DEFECTIVE-LIKE proteins were expressed in roots and were proposed as a positive regulator of root hair formation [37]. CRK is involved in hormone signalling and root growth in Arabidopsis [38] while RHD3 (a GTP-binding protein) has been associated with root development in poplar [39]. An miR171 family member (mac-miR171b) was down-regulated in the salt-stressed banana roots while two predicted targets, members of the GIBBERELLIN-INSENSITIVE, REPRESSOR of ga1-3 and SCARECROW (GRAS) family transcription factors, which also have been reported to control lateral root development [40], showed differential expression, one raised and the other down-regulated (Fig 4). These patterns of expression indicate that a reduced level of these miRNAs, permits increased levels of proteins required for root adaptation in response to high salinity. In the study on RNA-seq of white lupin [37] (mentioned above) members of the GRAS family transcription factors were also shown to be up-regulated in roots during development and thus regarded as a positive regulator of root hair formation. The regulation of root plasticity during stress remains a relatively poorly understood area of plant molecular biology that is critical for plant breeding and the development of abiotic stress resistant crops in the face of environmental degradation and climate change [5, 41]. The miRNA-target models identified in banana roots here, expand the potential pathways of miRNA regulation during salinity stress.

A second important aspect of plant responses to stress, is signal transduction and in this study we observed several signaling-related miRNA-target pairs that were differentially expressed in response to high salinity. These included transcription factor AP2 regulation by mac-miR172a.2 and mac-miR172f (Fig 4). AP2 domain-containing transcription factors have been reported to be a stress regulator with positive roles in abiotic stress responses in *Medicago sativa* and *Jatropha curcas* [42, 43] and are also up-regulated during salt stress in soybean roots [22]. Other potential targets of mac-miR172f which were up-regulated in the salinity stressed banana roots, included phospholipase D (PLD), which has been shown to enhance abiotic stress tolerance in plants [44], and a peroxisomal targeting signal. Several phospholipase D genes have been reported in an RNA-seq analysis of an oak species and were suggested to be involved in drought avoidance [45]. Plant peroxisomes have been reported to be involved in responses to abiotic and biotic stresses [46]. A predicted target of mac-miR397, osmotic stress-activated protein kinase, also up-regulated in the stressed banana root, has been reported to be involved in nitric oxide signaling and a hyperosmotic stress response in tobacco [47]. Similarly, a putative signal peptidase and a sialyl transferase (glycosyltransferase), other predicted targets of mac-miR397, were upregulated. Other signaling related miRNA-targets pairs in which the target mRNA was up-regulated in the stressed root samples include mac-miR529b with an ATP/GTP binding protein target and mac-miR535c with a putative G-type lectin S-receptor-like serine/threonine-protein kinase (GsSRK) target. GsSRK from soybean has been shown to be induced by salt stress and to improve plant tolerance to salt stress when heterologously expressed in Arabidopsis [48]. The increased levels of the several potential targets of banana miR172, miR397, miR529 and miR535, which were down-regulated during salinity stress, suggest an important network of regulation and signalling in the banana root, to enable multiple physiological responses.

Another salinity tolerance mechanism used by plants, is the synthesis of compatible solutes as a method of osmoprotection. The increased expression of dipeptidyl peptidase in conjunction with reduced expression of its predicted regulatory mac-miR162b.2 in the salt-stressed banana root both in the transcriptome data (Fig 4) and validated by RT-qPCR (Fig 5e), may reflect increased synthesis of the osmoprotective molecule proline as dipeptidyl peptidase has been suggested to degrade small proline-containing peptides in barley [49]. Also of interest were two mac-miR159 sequences, down-regulated in the salt-stressed banana roots while their predicted targets, salt responsive protein 2 and chorismate mutase, were up-regulated, both in the transcriptome (Fig 4) and RT-qPCR data (Fig 5c and 5d). Chorismate mutase catalyzes the early biosynthesis step of phenylalanine and tyrosine, the precursors to a number of secondary metabolites important for development and stress responses in Arabidopsis [50]. Salt responsive protein 2 has been described as an early salt stress response gene found in tomato root analyzed using suppression subtractive hybridization and microarray [51]. Several other studies have reported roles for miR159 in salt stress responses (Fig 6).

The current study identified a number of *Musa*-specific miRNA (i.e. with a qualifying precursor sequence in the banana genome, but no qualifying match to any known miRNA), several of which were differentially expressed in response to elevated salinity (Fig 4). This includes the mac-miR6 and its predicted dehydrin-domain carrying target (CL1Contig328), a predicted zinc finger CCCH domain-containing protein (target of mac-miR19), a chloride channel protein (target of mac-miR37), an auxin transport-associated protein SORTING NEXIN 1 (target of mac-miR49) and a DEAD-like helicase (target of mac-miR35 and mac-miR66). All of the predicted mRNA targets have been associated with salinity responding pathways, for example chloride channels function as both ion channel and transporter in plants [52]. In each case, the *Musa*-specific regulatory miRNA showed an inverse level of expression to that of its target mRNA in the salt-treated roots with the majority showing an increase in expression of target in the salinity-stressed root, as validated by RT-qPCR for all five miRNA and for their mRNA targets, with the exception of DEAD-like helicase (the predicted target of mac-miR66, Fig 5). Several differentially-expressed *Musa*-specific miRNA had predicted targets with no known function. Among these, only CL1Contig328, a predicted target of mac-miR6, had a matching domain annotation, which was for dehyrin. Dehydrins are LEAII-family proteins involved in plant responses to cold, drought and salinity, which all result in cell dehydration [53] and has been reported in a transcriptomic study of salt-stressed roots in rice [54] thus a role in the stressed banana root seems plausable.

In general, the targets that showed inverse expression pattern with their corresponding miRNAs differentially expressed in the 300 mM NaCl-stressed banana showed increased expression levels, while their regulating miRNA was repressed. The main functions were related to the physiological changes in root adaption, signaling and general stress responses expected to result from the experimental conditions. Several of the miRNA have multiple predicted targets and some transcripts, such as ATP/GTP-binding protein, NADP-GAPDH, CL2012Contig1 and CL7009Contig, are probably highly regulated as in this present study they were targeted by more than one miRNA family (Fig 4). Some hypothetical or unnamed proteins and unannotated genes were predicted as miRNA targets and showed inverse expression pattern with their corresponding salt stress-responsive miRNAs (Fig 4). They are CL2012Contig (target of mac-miR156 and mac-miR49), C111260 (target of mac-miR157), CL1Contig6497 (target of miR528), CL7009Contig1 (target of miR529), C102056 (target of mac-miR38) and CL7639 (target of mac-miR62). These miRNA targets are probably novel transcripts or proteins expressed in the banana roots in response to salinity stress and are of interest for future functional investigation. Many of the affected processes and pathways observed in banana in our current study, are also reported for other plant salt-stress transcriptomes, indicating that miRNA-regulated genes form a significant proportion of genes responding to salinity stress and highlighting the role of miRNAs in the regulation of responses to salt stress in plants. The predicted miRNA-mRNA regulatory models in this present study may provide clues for further investigations that can potentially lead to crop genetic improvement for enhanced tolerance to abiotic stresses. This may be through marker assisted breeding or molecular breeding approaches, for example, the overexpression of miRNA169 has been used to improve drought tolerance in tomato [55]. In addition to the plant orthologous and *Musa*-specific (‘novel’) miRNAs, the huge number of unannotated small RNA sequences in the three small RNA datasets is likely to include functional small RNAs such as siRNA with functions yet to be revealed.

### Conclusions

The banana response to salinity stress includes changes in expression levels of a large number of genes and miRNA, including several *Musa*-specific miRNA sequences and miRNA targets. Most of the miRNA respond differently to different levels of salinity, with a larger number repressed in roots exposed to 300mM NaCl. This likely reflects different programs of response for root adaptation or tissue necrosis for plant survival strategies, as the functions of many of the predicted miRNA targets relate to important pathways for signaling and physiological adaptation, in particular root development. To our knowledge, this is the first report of high-throughput genetic resources for an abiotic stress response in banana. The high-throughput sequencing data generated in this present study may serve as important genetic resources for salt-tolerance traits used for functional genomic studies and genetic improvement in banana.

## Materials and Methods

### Ethics

The conduct of this research was approved by the grant management committee of the University of Malaya, headed by the Director of Institute of Research Management and Monitoring, Professor Noorsaadah Abdul Rahman (noorsaadah@um.edu.my), and does not involve the use of any human, animal and endangered or protected plant species as materials.

### Plant materials and samples treatments

Clonal tissue culture derived banana plantlets, *Musa acuminata* cultivar Berangan (AAA triploid genome) possessed healthy roots were used in this study. Only plantlets sized 6–8 cm were selected for stress treatments. *In vitro* banana plantlets were treated on MS basal medium supplemented with 0 mM (CTR), 100 mM (TR100) and 300 mM (TR300) NaCl respectively for 48 hours prior to RNA isolation. Root tissues were pooled randomly from 3–4 plantlets in each treatment for RNA isolation.

### RNA isolation

RNA was isolated from banana roots using a CTAB-based RNA isolation method [56] with an additional extraction step using phenol-chloroform-isoamylalcohol (25:24:1). Absorbance at 260 nm and 280 nm was determined spectrophotometrically and the Agilent 2100 Bioanalyzer (Agilent Technologies Inc., Santa Clara, CA, USA) was used for determining RNA integrity. Only RNA samples with A260/A280 ratio ranged 1.8–2.2, A230/A260 ratio higher than 1 and RNA integrity number (RIN) higher than 8 were used for small RNA and transcriptomic libraries construction and sequencing using Illumina GAxII and HiSeq 2000 platforms (Illumina Inc., San Diego, CA, USA).

### Illumina paired-end cDNA library construction and sequencing (RNA-Seq)

RNA-Seq (RNA sequencing) and sRNA-Seq (small RNA sequencing) services were provided by BGI-Shenzhen, China. Two libraries were constructed from purified mRNA isolated from banana roots, namely CTR (control) and TR300 (300 mM NaCl treatment) and sequenced using Illumina HiSeq 2000 platform (Illumina Inc., San Diego, CA, USA) according to the manufacturer’s protocol (Illumina Inc., San Diego, CA, USA). In brief, Sera-mag Magnetic Oligo (dT) beads (Illumina Inc., San Diego, CA, USA) were used to isolate poly(A) mRNA from total RNA. Fragmentation buffer containing divalent cations was used to interrupt mRNA into short fragments. Random hexamer primers (Illumina Inc., San Diego, CA, USA) were used to synthesize the first-strand cDNA from these short fragments. The second-strand cDNA was synthesized in reaction buffer containing dNTPs, RNaseH and DNA polymerase I. Short fragments were then purified with QIAQuick PCR extraction kit (Qiagen, Hilden, Germany) and resolved with EB buffer for end repair and addition of poly(A). After that, the short fragments are connected with sequencing adapters. After the agarose gel electrophoresis, the suitable fragments were selected for the PCR amplification as templates. Lastly, the library was sequenced using Illumina HiSeq 2000. The insert of the library is approximately 200 bp and both ends of the libraries were sequenced. The raw reads were processed to remove adapter sequences, empty reads and low quality sequences (containing ambiguous sequences, ‘N’). High quality (HQ) clean reads were determined and selected from the RNA-Seq data and were used for further analysis. The short reads of RNA-seq have been deposited in NCBI’s Sequence Read Archive (SRA) database with accession numbers SRX535340 and SRX535341 for control and 300 mM NaCl treatment respectively.

### *De novo* transcriptome assembly

After removal of adapters and low quality sequences, the clean reads were assembled into contigs, scaffolds and unigenes using a short reads assembling program, SOAP*denovo* assembler version 1.05 (http://soap.genomics.org.cn/soapdenovo.html) [57]. The clean reads were assembled using the 63-mer version of SOAP*denovo* and a K-mer size of 51. The SOAP*denovo* assembler was used to combine sequence reads to form longer fragments without an ambigouos nucleotides (‘N’). The assembled sequences containing no ‘N’s are called contigs. Then SOAP*denovo* was used to connect and link the contigs into scaffolds. The unknown bases in the scaffolds were filled with ‘N's. Paired-end reads were used again for gap filling of scaffolds by using GapCloser v.12 for SOAPdenovo (http://soap.genomics.org.cn/soapdenovo.html) to obtain sequences with the least ‘N’s that could not be extended on either end. Such sequences are defined as unigenes. The assembled unigenes from both the transcriptomes CTR and TR300 were clustered together using TGI clustering tool 2.1 to form a set of non-redundant unigenes. After clustering, the *de novo* assembled unigenes were mapped to the reference *Musa* genome [18] using BWA version 0.6.1 [58], with mismatch score less than 3.

### Functional annotation of transcriptome

The *de novo* assembled unigenes were assigned with putative identities and/or functional annotations by similarity searches against the publicly available reference protein databases. Standalone BLASTX was carried out using NCBI’s BLASTALL tool for similarity searches against the NCBI non-redundant (nr) protein database (Dec 29, 2011, 4:42 PM, Version 4, with 16,785,757 sequences) with a stringent cut-off E-value of 1e^-10^. The unigenes were also searched against the Uni-Prot database (Feb 28, 2012, 7:17 PM, Version 4, with 534,242 sequences) using standalone BLASTX with the same cut-off E-value. Blast2GO program (http://www.blast2go.com/b2ghome) version 2.7 was used to assign Gene Ontology (GO, http://www.geneontology.org/) terms and Kyoto Encyclopedia of Genes and Genomes (KEGG, http://www.genome.jp/kegg/) pathways to the unigenes. Eukaryotic Orthologous Groups of Proteins (KOG) assignment was carried out using the NCBI’s BLASTALL tool.

### Transcripts quantification and differential gene expression in salt stress transcriptome

Library normalization was conducted prior to expression comparison of transcripts between the two libraries (CTR and TR300). Transcripts were normalized to transcript per million (TPM) [59]. An R package, DEGSeq (version 1.15, http://bioinfo.au.tsinghua.edu.cn/software/degseq) was used to identify differentially expressed unigenes based on binomial assumptions for the read count, using a multiple averaging (MA) plot based random sampling model (MARS) [60] as reported in [61–62]. The transcripts normalization and differential gene expression were calculated using an R script. Only unigenes with at least 10 read counts present in the library were used for transcript quantification. False Discovery Rate (FDR) was calculated using the DEGSeq package with Benjamini-Hochberg multiple test correction [63]. FDR ≤0.05 and log_2_ ratio equal to or higher than 1 were set as threshold values to select for differentially expressed transcripts.

### Illumina small RNA library construction and sequencing (sRNA-Seq)

Small RNA library construction for Illumina sequencing was carried out using Illumina’s kit according to the manufacturer’s recommendations. In brief, small RNAs of 16 to 28 nt were recovered from high resolution gel (15% (w/v) PAGE) and then ligated with 5’ and 3’ Illumina adapters using T4 RNA ligase. The small RNA-adapters ligation products were amplified by RT-PCR using Illumina’s small RNA primer set and the cDNA was sequenced using Illumina Genome Analyzer, GA IIx platform following the manufacturer’s instructions. The sequenced short reads have been deposited in NCBI’s Sequence Read Archive (SRA) database with accession numbers SRX535343, SRX535344 and SRX535345 for control, 100 mM and 300 mM NaCl treatments respectively.

### microRNA (miRNA) annotation

The Illumina small RNA sequence reads of banana root described in this study are available under NCBI’s BioProject accession PRJNA246442. Short Read Mapping Package (SHRiMP version 2.2.3, http://compbio.cs.toronto.edu/shrimp/) [64] was used to map the filtered reads to Rfam 11.0 (http://rfam.xfam.org/) [65] with no mismatch allowed. The small RNA sequences that matched with the non-protein-coding RNA sequences including rRNA, tRNA, small nuclear RNA (snRNA) and small nucleolar RNA (snoRNA) in Rfam were removed from further analysis. Sequences below 19 and above 24 nucleotides were also removed from the data set and not used in further analysis. The filtered reads were used to search for orthologous miRNAs. A non-redundant dataset was produced from the Plant microRNA Database (PMRD June 11, 2012 update, http://bioinformatics.cau.edu.cn/PMRD/) [66] and used as an orthologous plant miRNA reference set. A Python script was used to align the small RNAs with the reference set and only one mismatch was allowed for the alignment. The orthologous miRNA matches were named according to the original gene name except the species name, which was replaced with *Musa acuminata* (mac-).

After removal of sequences that matched to entries in the Rfam 11.0 and PMRD databases (June 11, 2012 update), the remaining small RNA sequences were used for the prediction of *Musa*-specific miRNAs, which were sequences not reported as miRNA in species other than *Musa* spp., from the *Musa* reference genome [18]. MiRDeep2 tool [67] was used and criteria for miRNA prediction from the plant genome were set according to [68]. The small RNA sequences were mapped to the reference A-genome [18] by using Bowtie [69] in the miRDeep2 pipeline. Using default parameters, three hundred nucleotides spanning the matched small RNA sequences in the reference genome were excised for stem-loop structure prediction using RNAfold [70]. Then, the p-values were calculated for the miRNA precursors predicted by miRDeep2 using Randfold [71]. The criteria used to annotate *Musa*-specific candidate miRNA were: (i) transcripts (unigenes) could form a stem-loop structure of 75-nt with a bulge-loop size less than 6 nt; (ii) small RNA reads fell within the stem region of the precursor; (iii) a maximum of 3 mismatches was allowed between the miRNA:miRNA* duplex; miRNA and miRNA* formed a duplex with 3’ overhangs; (iv) predicted minimum folding energy (MFE) was between -15 kcal/mol to -47.2 kcal/mol. *Musa*-specific miRNAs were arbitrarily named starting at ‘1’ and using the miRBase gene nomenclature [72].

### microRNA differential expression in sRNA-Seq data

MiRNA read counts were normalized to tags per million (TPM) in order to compare miRNA expression across libraries (CTR, TR100 and TR300). The TPM was calculated as follows: normalized expression, TPM = (actual miRNA count/total clean read) x 1,000,000. Differential miRNA expression was determined using DEGSeq [60] with absolute log_2_ Fold change equals to or higher than 1 and cut-off false discovery rate (FDR) value less than 0.05. Expression change was plotted in heatmaps using gplots package (http://cran.r-project.org/web/packages/gplots/index.html) from bioconductor in R.

### MicroRNA target prediction

The salt-stressed banana root transcriptomes (mRNA-Seq) in this study were used as reference data to predict miRNA targets using the psRNAtarget online server [73].

### Quantitative Real-Time RT-PCR

Reverse transcription and real-time PCR were performed to examine and validate expression pattern of 12 selected miRNAs and 14 target mRNAs. All primers involved in RT-qPCR were purchased from Integrated DNA Technologies (IDT) and the primer sequences were listed in Tables I–K in S1 File.

For miRNA, miRNA-specific stem-loop RT primers (Table I in S1 File) were designed and prepared based on a published method [74] and used for reverse transcription of the purified total RNA. Reverse transcription was performed according to a published method [75] using SuperScript III First-Strand Synthesis System (invitrogen, Life Technologies, Thermo Fisher Scientific Corporation, Waltham, MA, USA) and the cDNA was then used for real-time PCR using gene specific forward primers and universal reverse primer (Table J in S1 File).

For target mRNAs, genomic DNA elimination and reverse transcription were performed using QuantiTect Reverse Transcription Kit (QIAGEN, Hilden, Germany). Primers for real-time PCR were designed using PrimeQuest IDT (Integrated DNA technologies, https://sg.idtdna.com/PrimerQuest/Home/Index) (Table K in S1 File).

All real-time PCR analyses were carried out in a 20-μl reaction using Power SYBR Master Mix (Life Technologies, Thermo Fisher Scientific Corporation, Waltham, MA, USA) and performed on the QuantStudio 12K Flex real-time PCR platform (Applied Biosystems, Life Technologies, Thermo Fisher Scientific Corporation, Waltham, MA, USA). The reactions were incubated in 8-tube strips at 95°C for 10 min, followed by 40 cycles of 95°C for 15 s and 60°C for 1 minute. Dissociation curves for all target genes were established at the end of PCR cycle at 95°C for 15 s, 60°C for 1 minute, followed by 95°C for 15 s. The dissociation curves are shown in Fig I in S1 File. U6 was used as the reference gene. All reactions were run in triplicates for three biological replicates. The results were analyzed by Delta Delta C_T_ method.

## Supporting Information

S1 File Overview of the length distribution of the assembled contigs and unigenes (Figure A). Gap distribution in the assembled scaffolds and unigenes (Figure B). BLAST hits of the *de novo* assembled unigenes (Figure C). Eukaryotic Orthologous Group (KOG) annotation (Figure D). KEGG pathway assignment (Figure E). Length distribution of clean reads in small RNA libraries (Figure F). Classification of small RNA using PMRD and Rfam databases as reference (Figure G). Gene Ontology (GO) assignment for targets of the differentially expressed miRNAs in salt-stressed banana roots (Figure H). Dissociation curves of RT-qPCR for selected orthologous microRNAs (a-f) and *Musa*-specific microRNAs (g-l) (Figure I). Dissociation curves of RT-qPCR for selected target mRNAs (Figure J). Paired-end transcriptome sequencing (RNA-Seq) output (Table A). *De novo* assembly of banana root transcriptomes (Table B). Coverage of the assembled transcriptomes (Table C). Mapping of the *de novo* assembled unigenes to the reference A-genome (Table D). Statistics of small RNA sequence reads (Table E). Annotation of orthologous miRNAs in banana root sRNAomes (Table F). Putative *Musa*-specific miRNAs in banana root sRNAomes (Table G). Functions of predicted salinity-responsive miRNA / mRNA targets in banana roots (Table H). Stem-loop (SL) primers used for reverse transcription (RT) of microRNAs (Table I). Primers used for real-time RT-qPCR analyses of microRNAs (Table J). Primers used for real-time RT-qPCR analyses of target mRNAs (Table K).
(DOCX)
