# Supplementary material for: Transcripts and MicroRNAs Responding to Salt Stress in Musa acuminata Colla (AAA Group) cv. Berangan Roots
Source: PLoS One. 2015 May 20;10(5):e0127526. doi: 10.1371/journal.pone.0127526 (PMC4439137; doi:10.1371/journal.pone.0127526)
Supplement: S1 File — Gap distribution in the assembled scaffolds and unigenes (Figure B). BLAST hits of the de novo assembled unigenes (Figure C). Eukaryotic Orthologous Group (KOG) annotation (Figure D). KEGG pathway assignment (Figure E). Length distribution of clean reads in small RNA libraries (Figure F). Classification of small RNA using PMRD and Rfam databases as reference (Figure G). Gene Ontology (GO) assignment for targets of the differentially expressed miRNAs in salt-stressed banana roots (Figure H). Dissociation curves of RT-qPCR for selected orthologous microRNAs (a-f) and Musa-specific microRNAs (g-l) (Figure I). Dissociation curves of RT-qPCR for selected target mRNAs (Figure J). Paired-end transcriptome sequencing (RNA-Seq) output (Table A). De novo assembly of banana root transcriptomes (Table B). Coverage of the assembled transcriptomes (Table C). Mapping of the de novo assembled unigenes to the reference A-genome (Table D). Statistics of small RNA sequence reads (Table E). Annotation of orthologous miRNAs in banana root sRNAomes (Table F). Putative Musa-specific miRNAs in banana root sRNAomes (Table G). Functions of predicted salinity-responsive miRNA / mRNA targets in banana roots (Table H). Stem-loop (SL) primers used for reverse transcription (RT) of microRNAs (Table I). Primers used for real-time RT-qPCR analyses of microRNAs (Table J). Primers used for real-time RT-qPCR analyses of target mRNAs (Table K). (DOCX) [file pone.0127526.s001.docx]

**Supporting Information (SI)**

**A - contigs**

**
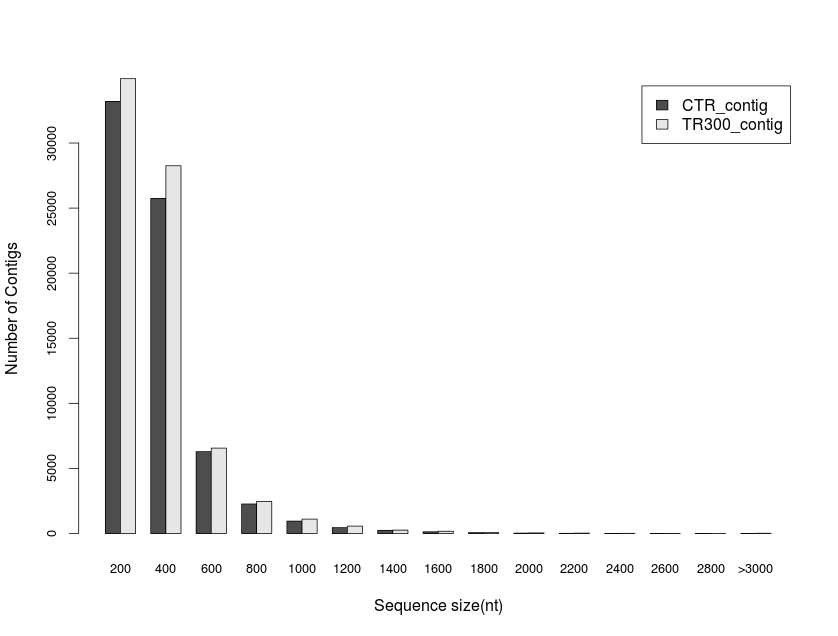
**

**B - unigenes**

**
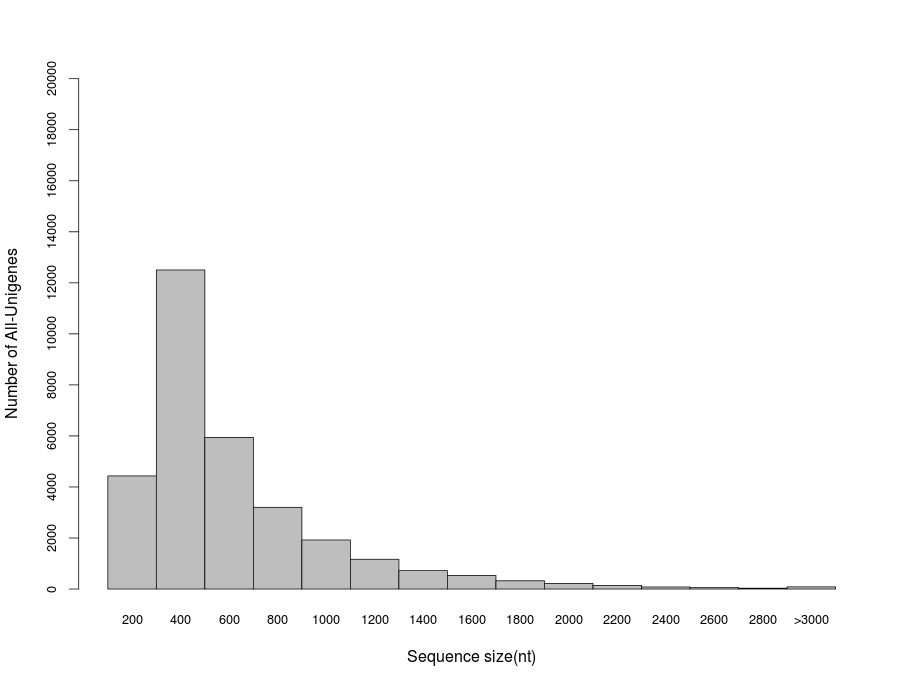
**

**Figure A Overview of the length distribution of the assembled contigs and unigenes. A.** Contigs in CTR and TR300, **B.** The assembled unigenes from both CTR and TR300 (All Unigenes)

**
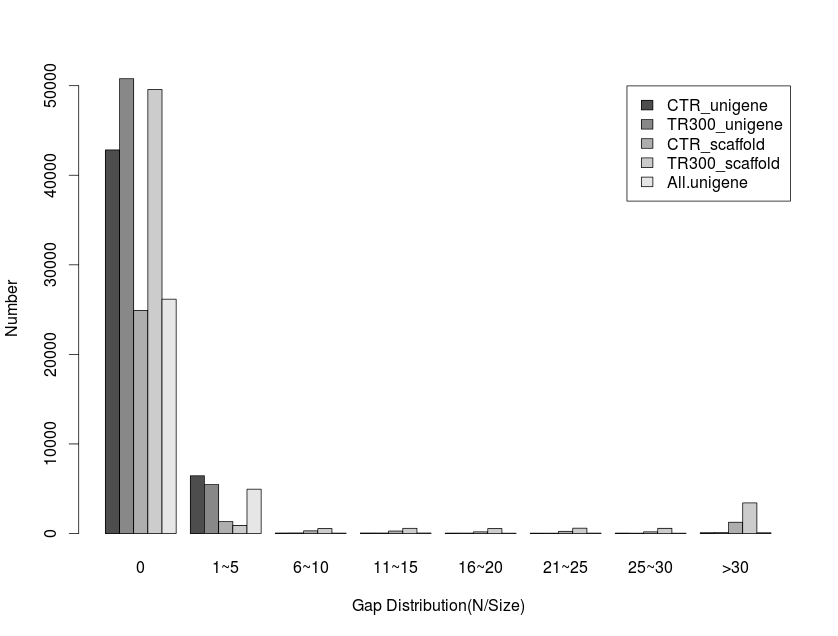
**

**Figure B Gap distribution in the assembled scaffolds and unigenes.** (N/size) % = percentage of ambiguous nucleotide (‘N’) in a sequence


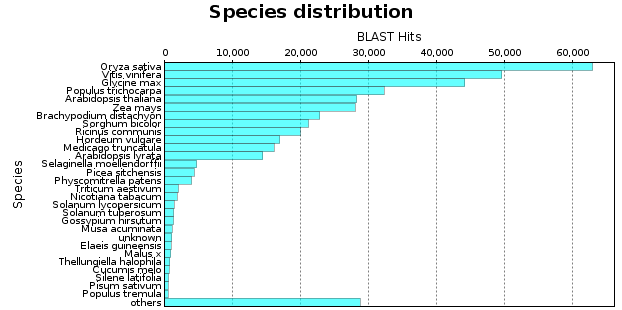


**Figure C BLAST hits of the *de novo* assembled unigenes.** Assembled unigenes from banana root transcriptomes searched against sequences comprised of different plant species deposited in the GenBank non-redundant protein database


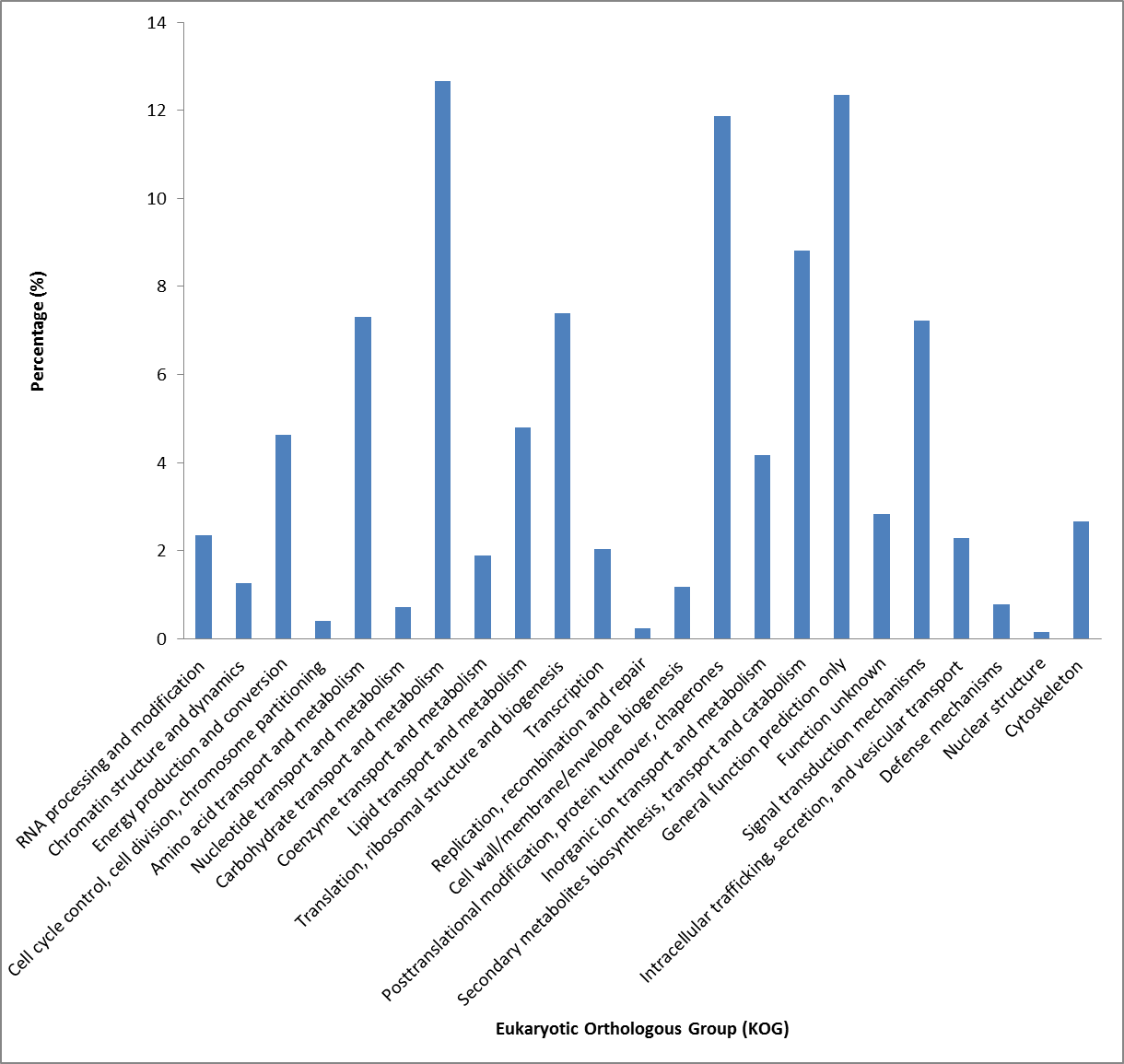


**Figure D Eukaryotic Orthologous Group (KOG) annotation.** Assignment of KOG to the differentially-expressed unigenes in salt-stressed banana roots


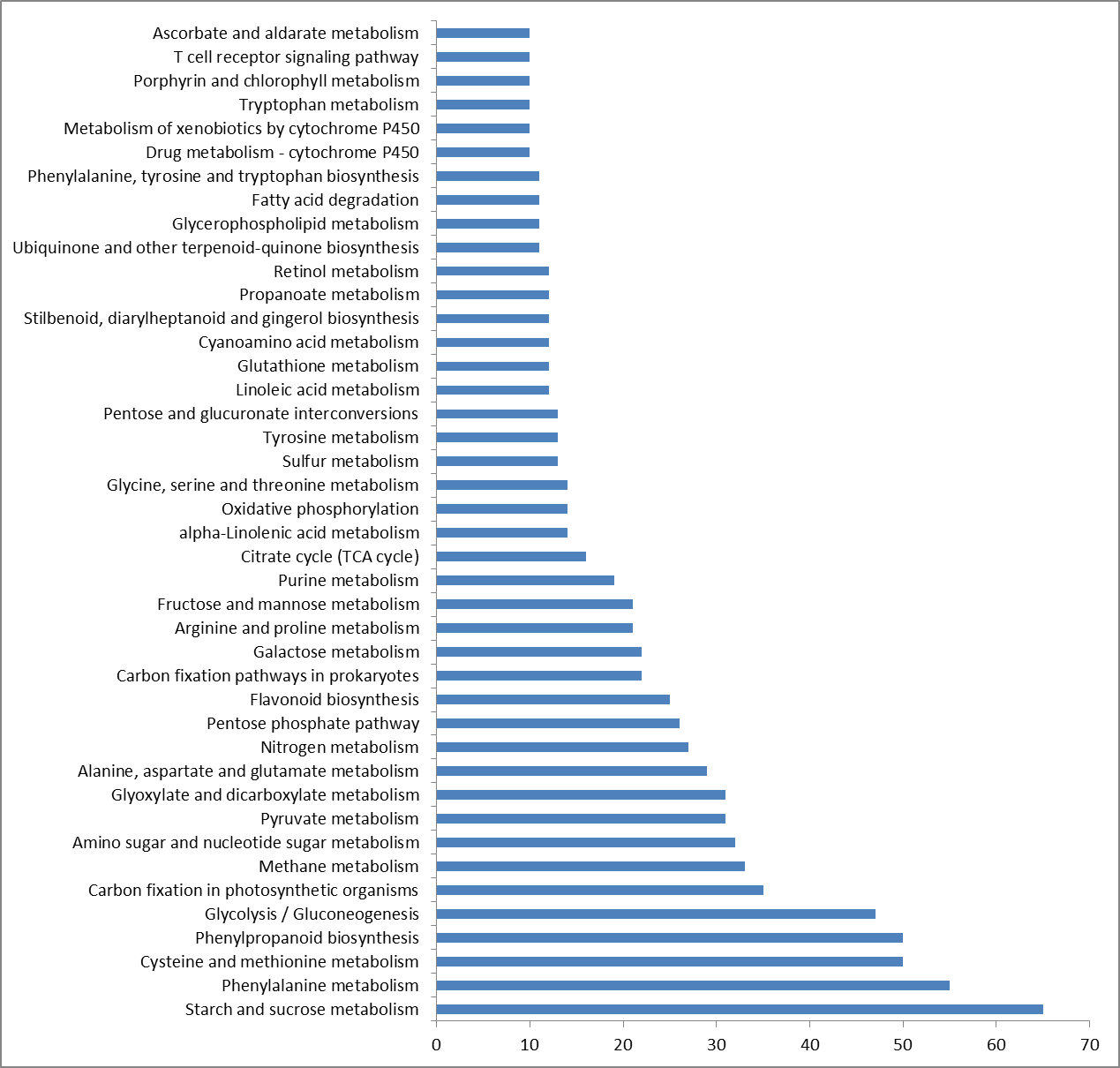


Number of unigene

KEGG pathways

**Figure E KEGG pathway assignment.** Differentially expressed unigenes in salt-stressed banana roots were assigned with KEGG pathways. Only KEGG pathways with at least 10 unigenes assigned are presented

Read count

Read length (nt)

Read count

Read length (nt)

**Figure F Length distribution of clean reads in small RNA libraries.** Sequence with length 21-nt is the most abundant one in all the three data set, followed by 24-nt and 20-nt (with exception in TR300 which 20-nt is the second and 24-nt the third most abundant sequences). CTR: untreated control (0 mM NaCl), TR100: 100 mM NaCl-treated banana roots; TR300: 300 mM NaCl-treated banana roots

**B** - TR100

**Figure G Classification of small RNA using PMRD and Rfam databases as reference**

**C** - TR300

**Figure G Continued**


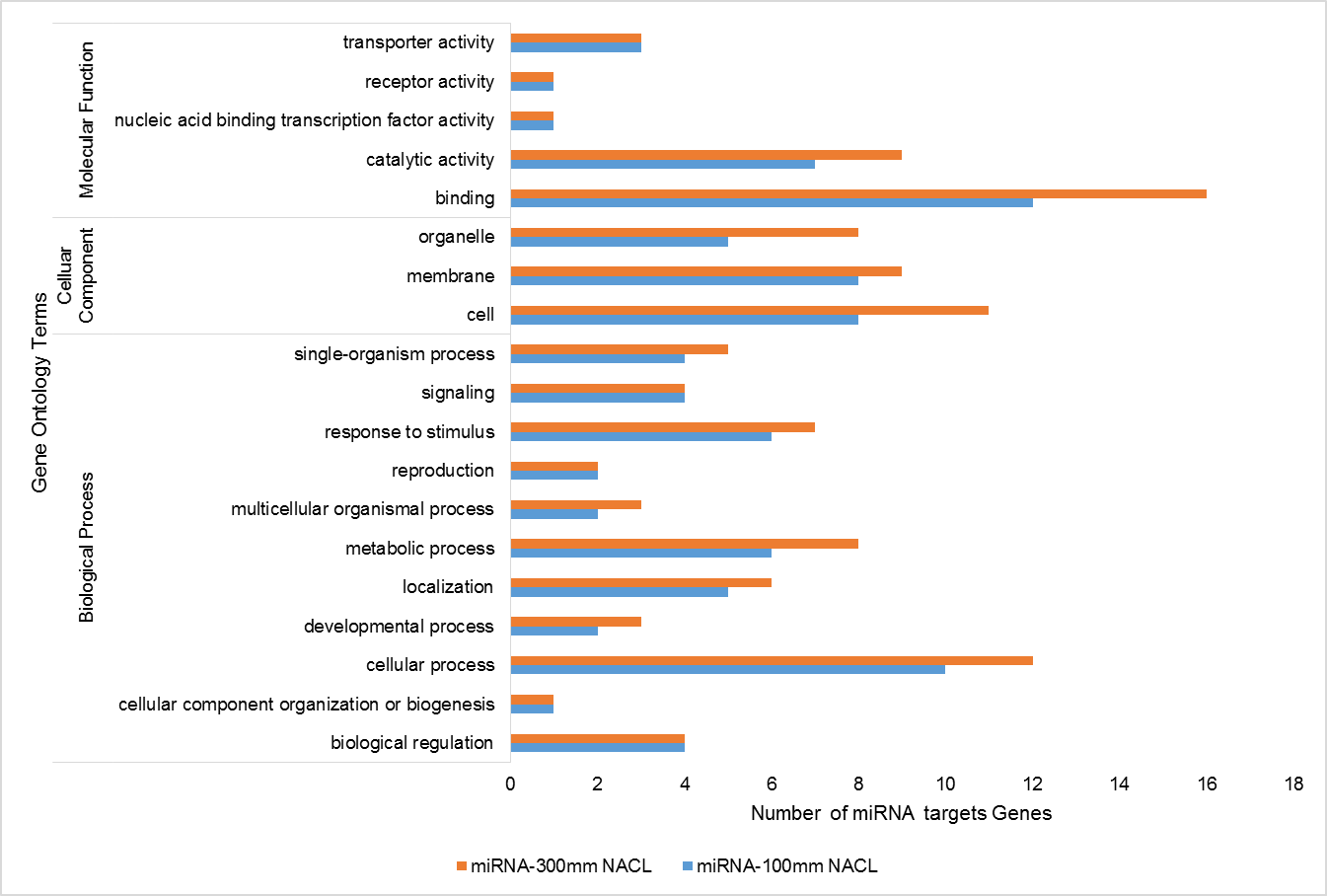


**Figure H Gene Ontology (GO) assignment for targets of the differentially expressed miRNAs in salt-stressed banana roots**

| (a) mac-miR156  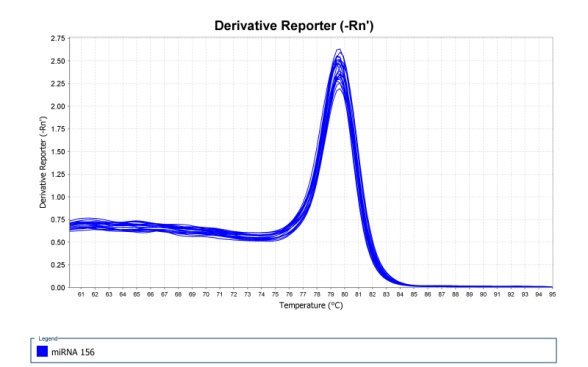 | (d) mac-miR157m  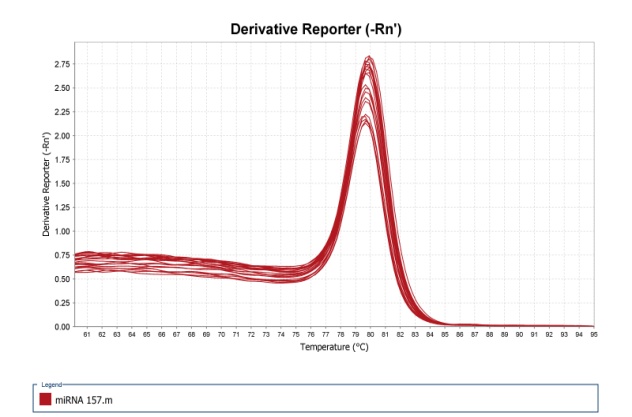 |
| --- | --- |
| (b) mac-miR159c  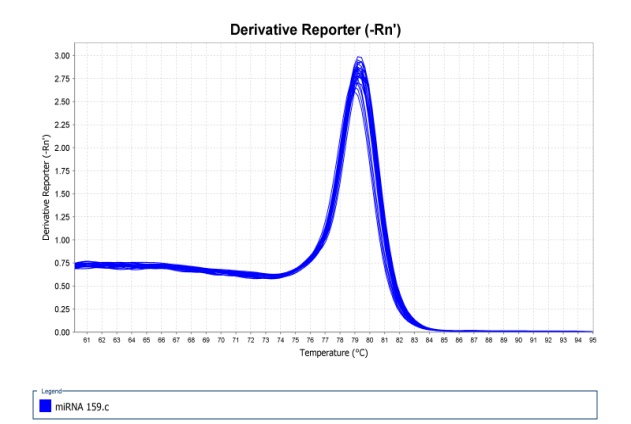 | (e) mac-miR162b.2  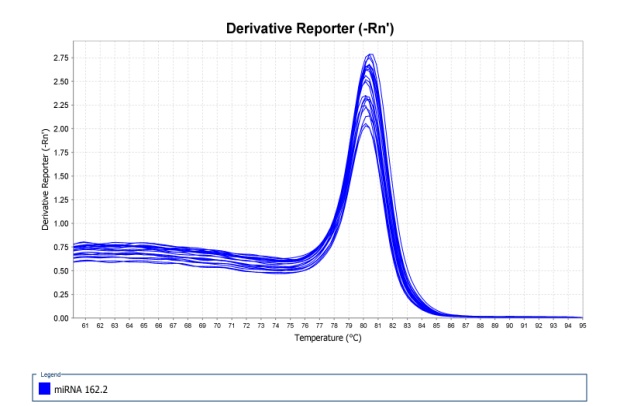 |
| (c) mac-miR159g  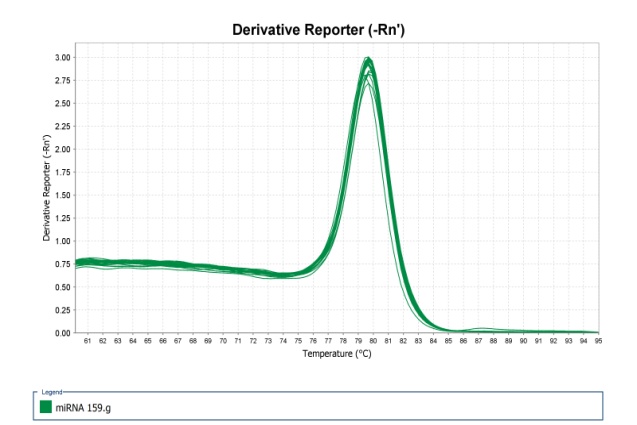 | (f) mac-miR168  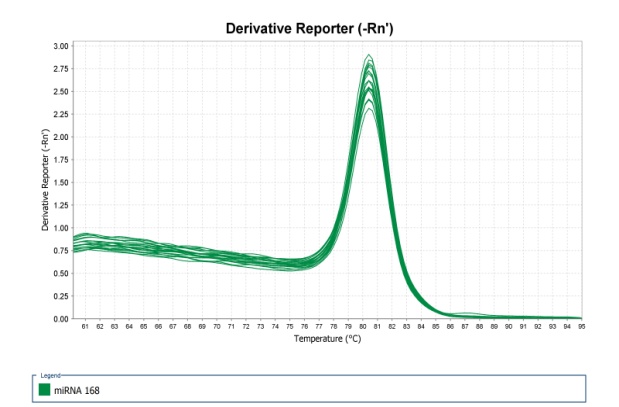 |

**Figure I Dissociation curves of RT-qPCR for selected orthologous microRNAs (a-f) and *Musa*-specific microRNAs (g-l)**

| (g) mac-miR6  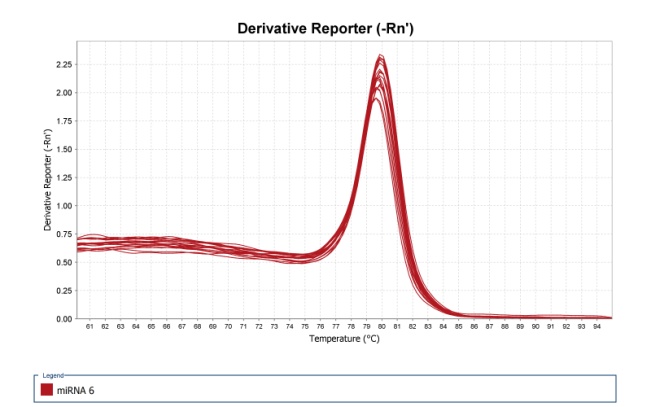 | (j) mac-miR49  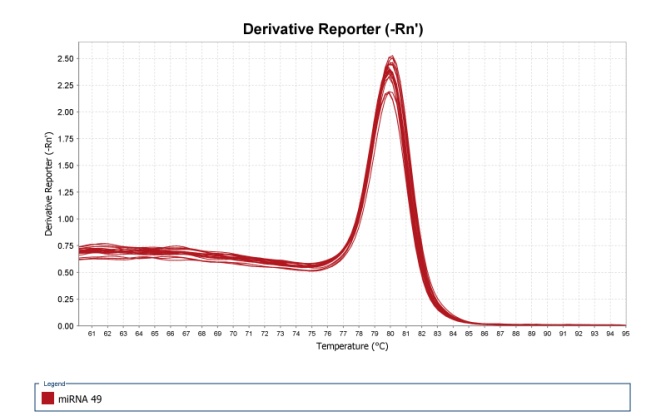 |
| --- | --- |
| (h) mac-miR19  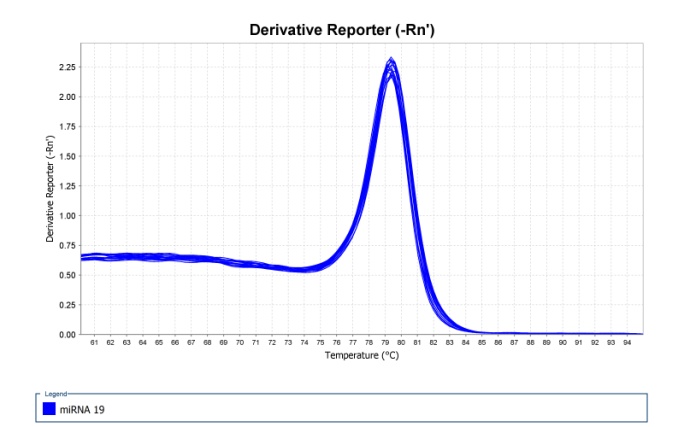 | (k) mac-miR62  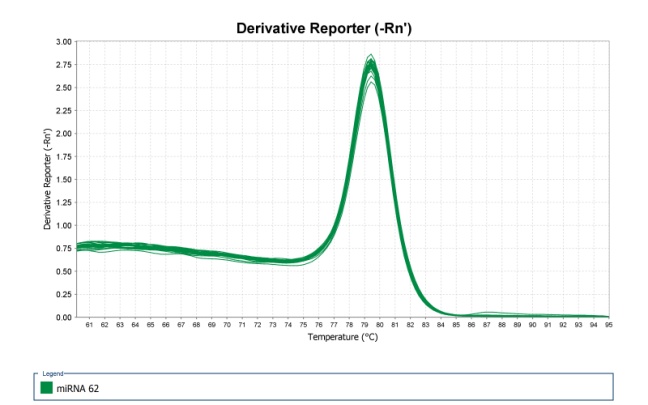 |
| (i) mac-miR37  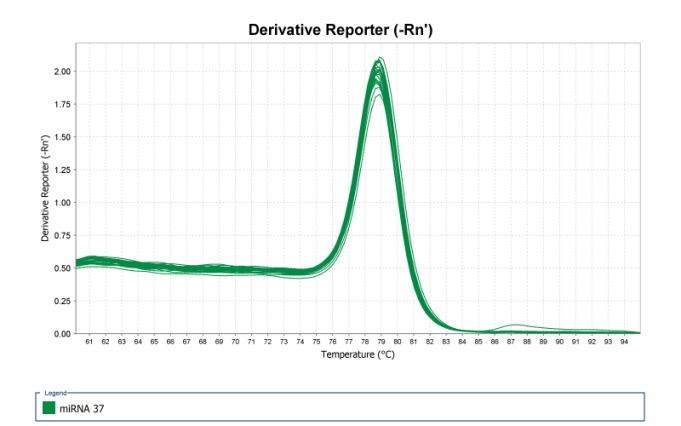 | (l) mac-miR66  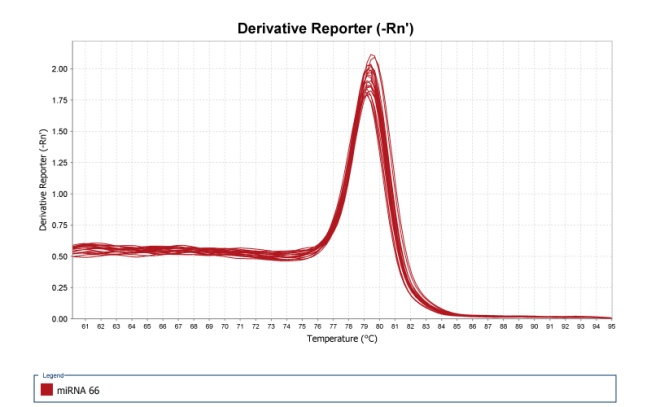 |

**Figure I Continued**

| (a) C137988_Tropine dehydrogenase_1b  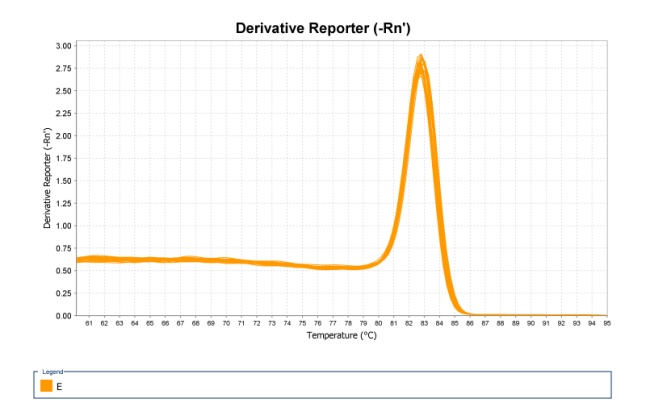 | (d) CL1Contig328_Dehydrin_32a  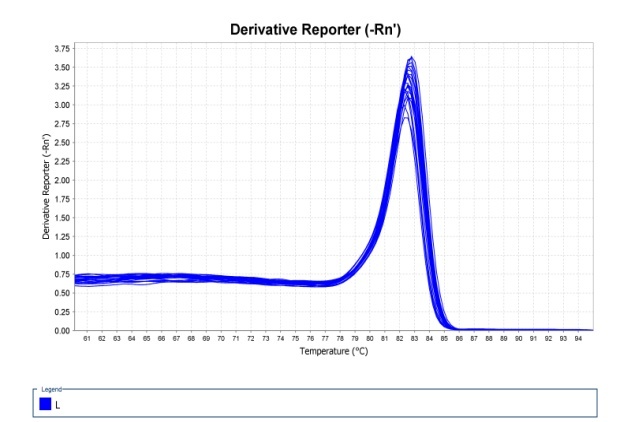 |
| --- | --- |
| (b) C78790_Avr Cf9 rapidly elicited_1c  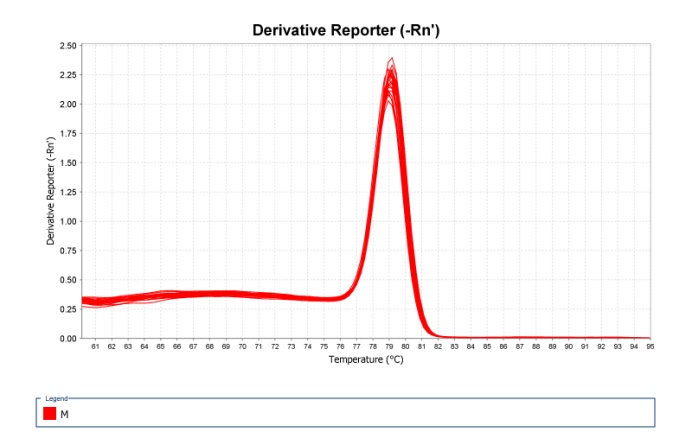 | (e) CL8970Contig1_Zinc finger CCCH domain containing protein_36a  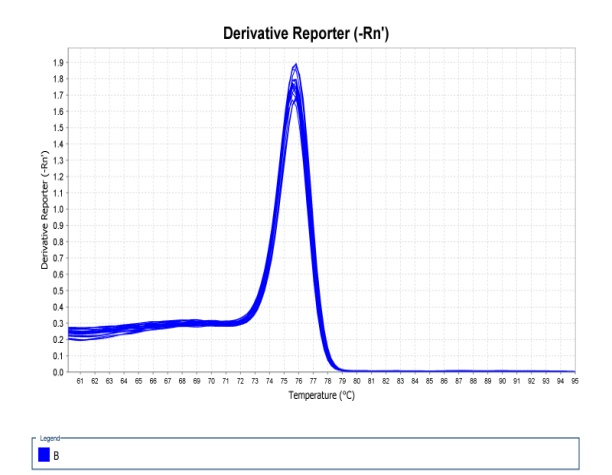 |
| (c) CL1Contig2785_Chorismate mutase_12a  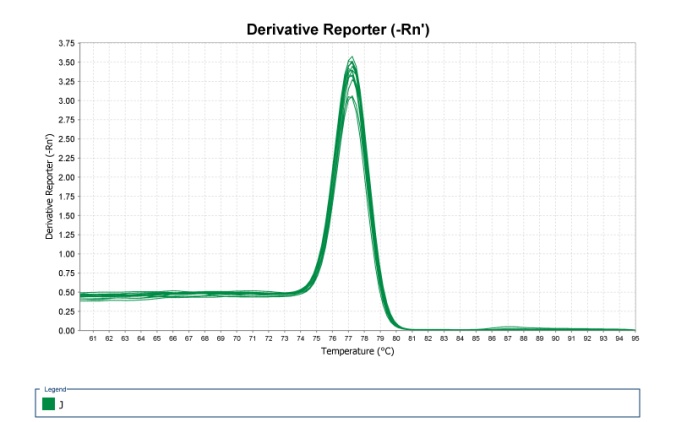 | (f) CL8325Contig1_Chloride channel_38a  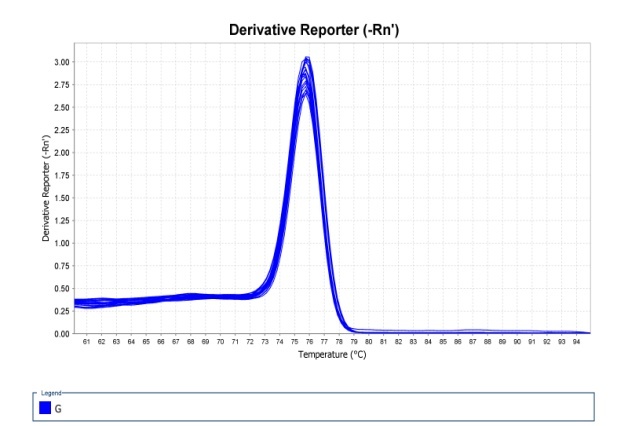 |

**Figure J Dissociation curves of RT-qPCR for selected target mRNAs**

| (g) CL8842Contig1_Salt responsive protein 2_13a  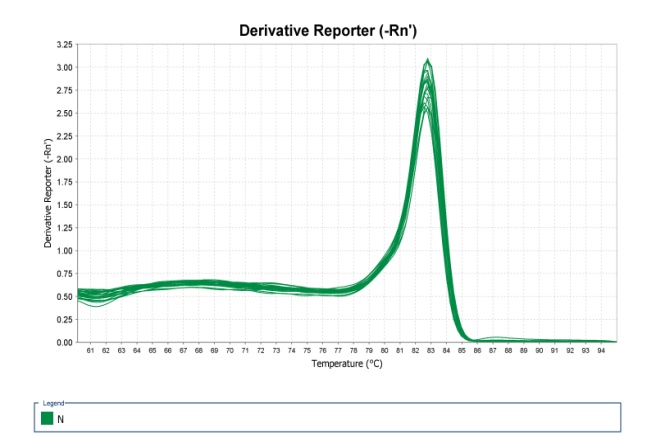 | (j) CL1Contig7021_Sorting nexin 1_40c  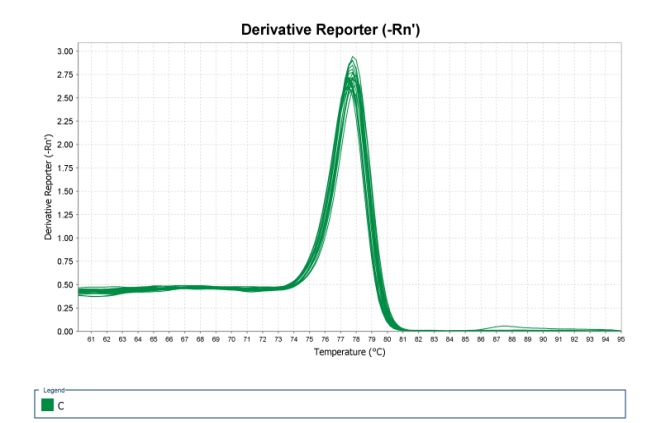 |
| --- | --- |
| (h) C133744_Protein root hair_defective3_11c  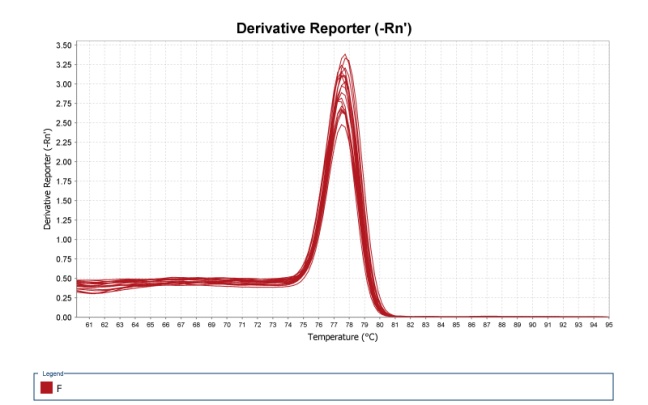 | (k) CL4298Contig1_Ubiquitin carboxyl-terminal hydrolase_40d  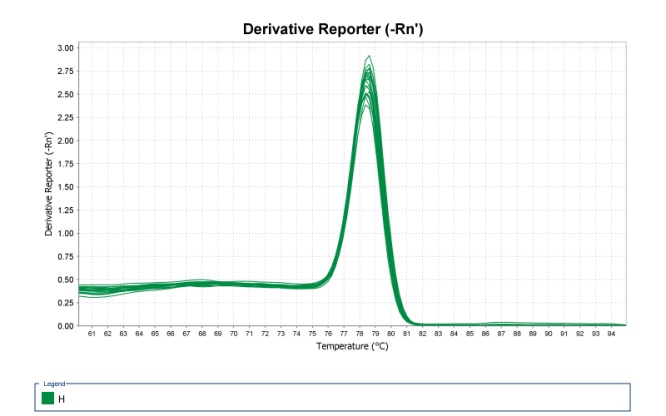 |
| (i) C71750_Dipeptidyl peptidase_14a_33a  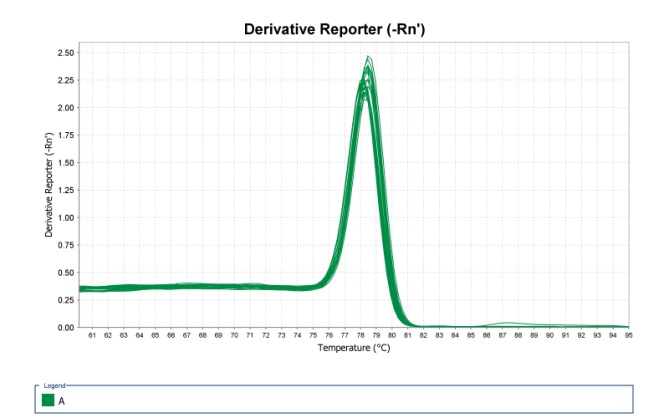 | (l) Scaffold8176_Protein IQ domain 1 like_42b  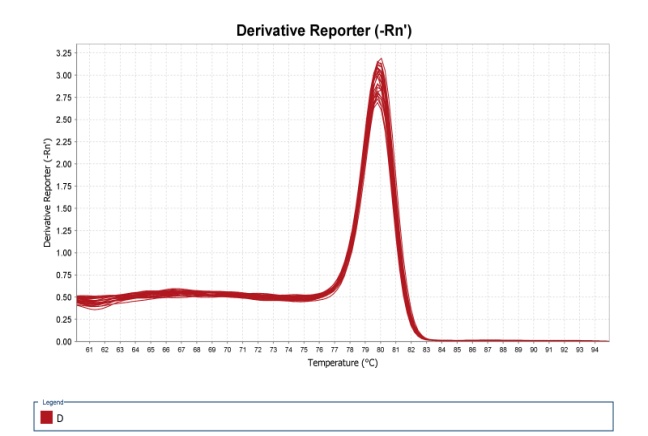 |

**Figure J Continued**

| (m) CL1Contig5779_12-oxophytodienoate reductase_18a  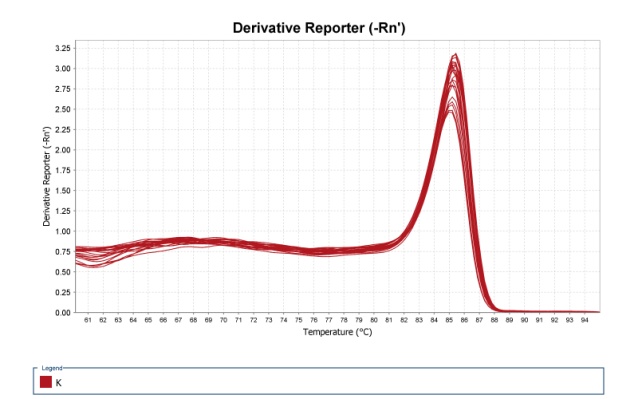 |
| --- |
| (n) C95248_DEAD like helicase_37a_43a  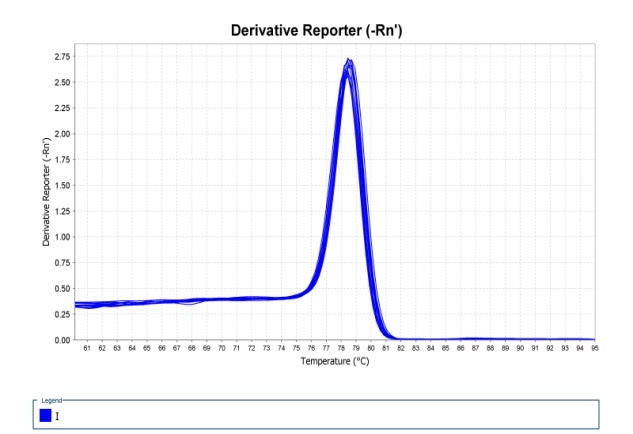 |
| (o) U6  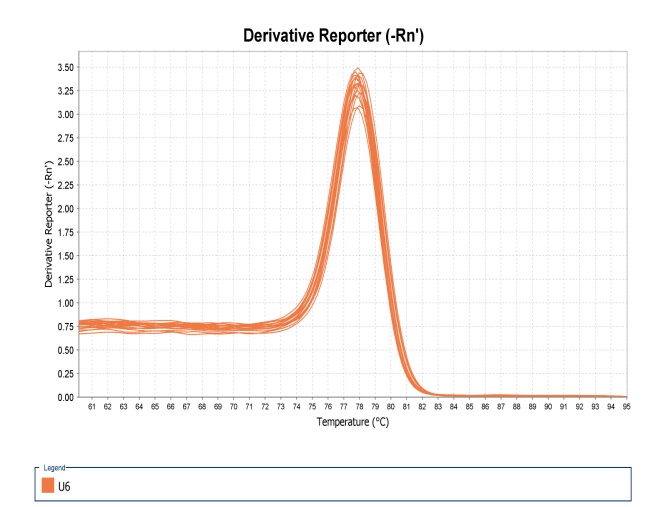 |

**Figure J Continued**

**Table A Paired-end transcriptome sequencing (RNA-Seq) output**

|  | **CTR** | **TR300** |
| --- | --- | --- |
| Total number of reads | 12,279,060 | 11,274,596 |
| Average read length | 90 | 90 |
| Total number of HQ reads* | 11,352,903 | 10,504,837 |
| Percentage of HQ reads | 92.46% | 93.17% |
| Total number of bases | 1,105,115,400 | 1,014,713,640 |
| Total number of HQ bases** | 1,037,037,224 | 956,651,654 |
| Percentage of HQ Bases | 93.84% | 94.28% |

CTR: untreated control (0 mM NaCl); TR300: 300 mM NaCl-treated banana roots; HQ: high quality

* >70% of bases in a read with Phred Quality Score > 20

** Bases with Phred Quality Score > 20

**Table B *De novo* assembly of banana root transcriptomes**

|  | **CTR** | | | **TR300** | | | **All** | | |
| --- | --- | --- | --- | --- | --- | --- | --- | --- | --- |
| Category | Total  count | Mean length (nt) | N50  (nt) | Total  count | Mean length  (nt) | N50  (nt) | Total  count | Mean length  (nt) | N50  (nt) |
| Contigs | 69,441 | 263 | 315 | 74,525 | 265 | 313 | - | - | - |
| Scaffolds | 49,576 | 349 | 441 | 56,572 | 334 | 411 | - | - | - |
| Unigenes | 49,576 | 350 | 443 | 56,572 | 334 | 411 | 31,390 | 517 | 669 |

CTR: untreated control (0 mM NaCl); TR300: 300 mM NaCl-treated banana roots; All: clustering of both CTR and TR300

**Table C Coverage of the assembled transcriptomes**

| **Data Set** | **Number of Reads*** | **Number of Bases**** | **Coverage** |
| --- | --- | --- | --- |
| **CTR** | **8,738,948** | **17,350,046** | 45.33X |
| **TR300** | **8,099,430** | **18,899,141** | 38.57X |

CTR: untreated control (0 mM NaCl); TR300: high salinity-stressed (300 mM) banana roots

*Number of reads that can be assembled into unigenes

**Number of bases in the assembled transcriptome

**Table D Mapping of the *de novo* assembled unigenes to the reference A-genome**

| **Chromosome** | **Number** | **Percentage (%)** |
| --- | --- | --- |
| 1 | 2,552 | 8.13 |
| 2 | 2,021 | 6.44 |
| 3 | 2,886 | 9.2 |
| 4 | 2,848 | 9.08 |
| 5 | 2,594 | 8.27 |
| 6 | 3,204 | 10.21 |
| 7 | 2,545 | 8.11 |
| 8 | 3,026 | 9.65 |
| 9 | 2,608 | 8.31 |
| 10 | 2,732 | 8.71 |
| 11 | 2,269 | 7.23 |
| Unrandom | 1,965 | 6.26 |
| Mapped | 31,250 | 99.56 |
| Unmapped | 140 | 0.54 |

**Table E Statistics of small RNA sequence reads**

| **Type** | **Read count** | | |
| --- | --- | --- | --- |
|  | **Control** | **TR100** | **TR300** |
| Raw reads | 18,189,390 | 18,396,868 | 19,641,136 |
| High quality reads | 15,911,422 | 16,039,100 | 15,190,814 |
| Clean reads* | 14,420,971 | 14,747,201 | 13,560,353 |
| Reads after collapse** | 3,438,498 | 3,302,845 | 1,539,695 |
| Reads aligned to genome*** | 1,910,703 | 1,749,569 | 910,246 |

*After filtering of contaminant sequences including sequencing adapters and poly(A)

**Non-redundant reads

***Non-redundant reads mappable to the reference *Musa* genome (D’Hont et al., 2012)

**Table F Annotation of orthologous miRNAs in banana root sRNAomes**

|  | **CTR** | **TR100** | **TR300** | **All (non-redundant)** |
| --- | --- | --- | --- | --- |
| Known miRNA | 153 | 149 | 128 | 181 |
| miRNA families | 39 | 40 | 35 | 47 |
| No. of miRNA targets | 110 | 115 | 91 | 274 |

CTR: untreated control (0 mM NaCl); TR100: 100 mM NaCl-treated banana roots; TR300: 300 mM NaCl-treated banana roots

**Table G Putative *Musa*-specific miRNAs in banana root sRNAomes**

|  | **CTR** | **TR100** | **TR300** | **All (non-redundant)** |
| --- | --- | --- | --- | --- |
| Novel miRNA | 47 | 40 | 42 | 56 |
| No. of miRNA targets | 67 | 81 | 66 | 120 |

CTR: untreated control (0 mM NaCl); TR100: 100 mM NaCl-treated banana roots; TR300: 300 mM NaCl-treated banana roots

**Table H Functions of predicted salinity-responsive miRNA / mRNA targets in banana roots**

| **Biological role** | **miRNA family** | **Target mRNA** | **ID** | **Target gene function** |
| --- | --- | --- | --- | --- |
| Root development | miR157 | CDPK-related kinase | CL3118Contig1 | hormone signaling and root growth in Arabidopsis [1] |
|  |  | Root hair defective 3 | C133744 | GTP-binding protein associated with root development in poplar [2] |
|  | miR171 | GRAS family transcription factor | CL7706Contig1 | controls lateral root development in Arabidopsis [3] |
| Development (reproduction) | miR397 | Sialyl transferase | CL1Contig5101 | pollen tube growth and pollen germination in Arabidopsis and rice [4, 5] |
|  | miR2910 | Cleavage and polyadenylation specificity factor | CL1Contig6924 | reproductive development [6] and immune response [7] in Arabidopsis |
| Signaling / Signal transduction | miR168 | 12-oxophytodienoic acid reductase | CL1Contig57790 | involved in biosynthesis of jasmonic acid, salinity tolerance in wheat [8] |
|  | miR172 | AP2 domain-containing transcription factor | CL5762Contig1 | abiotic stress responses in *Medicago sativa* and *Jatropha curcas* [9] [10] |
|  |  | Phospholipase D | CL1Contig2487 | enhanced abiotic stress tolerance in Arabidopsis [11] |
|  |  | Peroxisomal-targeting signal | CL1Contig7365 | involved in responses to abiotic and biotic stresses [12] |
|  | miR397 | Osmotic stress-activated kinase | CL1Contig6498 | nitric oxide signaling and hyperosmotic stress response in tobacco [13] |
|  |  | Signal peptidase | CL1Contig7326 | maturation of membrane proteins including signal peptides in Arabidopsis [14] |
|  | miR529 | ATP/GTP-binding protein | Contig431 | Signaling, defence, growth and development in plants [15, 16] |
|  | miR535 | G-type lectin S-receptor-like serine/threonine-protein kinase | Scaffold5199 | improved salt tolerance when heterologously expressed in Arabidopsis [17] |
|  | mac-miR19 | Cysteine3Histidine-type zinc finger protein | CL8970Contig1 | mRNA processing; plant growth, development, and stress response [18] |
|  | mac-miR38 | NADP-dependent GAPDH | C126430 | Stress signaling in ROS pathway [19, 20] |
|  | mac-miR49 | SORTING NEXIN 1 | CL1Contig7021 | Root cell endosomal auxin transporting efflux carrier PIN2 [21] |
|  | mac-miR62 | Protein IQ-domain | Scaffold8176 | associated with Ca^2+^ sensors /calmodulin to regulate at transcriptional and post-transcriptional level [22] |

**Table H Continued**

| **Biological role** | **miRNA family** | **Target mRNA** | **ID** | **Target gene function** |
| --- | --- | --- | --- | --- |
| Stress mechanisms | miR162 | Dipeptidyl peptidase | C71750 | Degrades small proline-containing peptides in barley [23] – synthessis of compatible solute |
|  | miR159 | Chorismate mutase | CL1Contig2785 | biosynthesis of amino acid precursors of secondary metabolites important for development and stress responses in Arabidopsis [24] |
|  |  | Salt responsive protein 2 | CL8842Contig1 | early salt stress response in tomato root [25] |
|  | miR528 | Leucyl-tRNA synthetase | scaffold4656 | aminocylation of tRNA for leucine [26] |
|  | mac-miR37 | Chloride channel | CL8325Contig1 | ion channel and transporter in plants [27] |
|  | mac-miR49 | Ubiquitin carbonyl-terminal hydrolase | CL4298Contig1 | deubiquitination of polyubiquitin precursors and ubiquitinated proteins [28] |
| Stress / defence | miR156 | Tropine dehydrogenase | C137988 | tropane alkaloid biosynthesis (herbivore defence) [29] |
|  | miR156 /miR157 | Avr/cf-9 rapidly elicited | C78790 | fungal and viral defence responses [30, 31] |
|  | miR397 | Laccase 110a | CL1Contig6779 | PAL / lignin pathway [32, 33] |
|  | miR528 | Polyphenol oxidase | CL1Contig337 | oxidation of ortho-diphenols to ortho-quinones important for defence in plants [34] |
|  | mac-miR6 | Dehydrin domain containing contig | CL1Contig328 | plant protective reaction to dehydration / osmotic stress [35] |
|  | mac-miR35 &  mac-miR66 | DEAD-like helicase | C95248 | effector RNA binding proteins in regulation of salinity stress response [36] |

**Table I Stem-loop (SL) primers used for reverse transcription (RT) of microRNAs**

| **Primer name** | **Primer sequence** |
| --- | --- |
| mac-miR6 SL RT Primer | GTC GTA TCC AGT GCA GGG TCC GAG GTA TTC GCA CTG GAT ACG ACAAGCCG |
| mac-miR19 SL RT Primer | GTC GTA TCC AGT GCA GGG TCC GAG GTA TTC GCA CTG GAT ACG ACGTTGGT |
| mac-miR37 SL RT Primer | GTC GTA TCC AGT GCA GGG TCC GAG GTA TTC GCA CTG GAT ACG ACTCAGAT |
| mac-miR49 SL RT Primer | GTC GTA TCC AGT GCA GGG TCC GAG GTA TTC GCA CTG GAT ACG ACGTGCTC |
| mac-miR62 SL RT Primer | GTC GTA TCC AGT GCA GGG TCC GAG GTA TTC GCA CTG GAT ACG ACAGGCTG |
| mac-miR66 SL RT Primer | GTC GTA TCC AGT GCA GGG TCC GAG GTA TTC GCA CTG GAT ACG ACGACCAA |
| mac-miR156 SL RT Primer | GTC GTA TCC AGT GCA GGG TCC GAG GTA TTC GCA CTG GAT ACG ACTGTGCT |
| mac-miR157m SL RT Primer | GTC GTA TCC AGT GCA GGG TCC GAG GTA TTC GCA CTG GAT ACG ACGTGCTC |
| mac-miR159c SL RT Primer | GTC GTA TCC AGT GCA GGG TCC GAG GTA TTC GCA CTG GAT ACG ACTAGAGC |
| mac-miR159g SL RT Primer | GTC GTA TCC AGT GCA GGG TCC GAG GTA TTC GCA CTG GAT ACG ACAGGAGC |
| mac-miR162b.2 SL RT Primer | GTC GTA TCC AGT GCA GGG TCC GAG GTA TTC GCA CTG GAT ACG ACCCGGAT |
| mac-miR168 SL RT Primer | GTC GTA TCC AGT GCA GGG TCC GAG GTA TTC GCA CTG GAT ACG ACTTCCCG |

Underlined: 6-nt sequence specific to target miRNAs

**Table J Primers used for real-time RT-qPCR analyses of microRNAs**

| **Primer name** | **Primer sequence** |
| --- | --- |
| mac-miR6_Forward | GCG GCG TAG GAG AGA TGA CA |
| mac-miR19_Forward | GCG GCT TCC AGG AGA GAT GA |
| mac-miR37_Forward | TAT GCG TAA AGC TGC CAG CA |
| mac-miR49_Forward | GAG GCC CTG ACA GGA GAG AGT |
| mac-miR62_Forward | GCG GCG AGA AGA GAG AGA GT |
| mac-miR66_Forward | TAT CCG GCG GGG TAG AGG AA |
| mac-miR156_Forward | GCG GCG TGA CAG AAG AGA GT |
| mac-miR157m_Forward | GCG GCG TTG ACA GAA GAG AG |
| mac-miR159c_Forward | GCG GCG TTT GGA TTG AAG GG |
| mac-miR159g_Forward | GCG GAG CTT GGA TTG AAG GG |
| mac-miR162b.2_Forward | GCG GCG TCG ATA AAC CTC TG |
| mac-miR168_Forward | GAT GAG TCG CTT GGT GCA GG |
| Universal stem-loop reverse primer | CCA GTG CAG GGT CCG AGG TA |

**Table K Primers used for real-time RT-qPCR analyses of target mRNAs**

| **Primer name** | **Primer sequence** |
| --- | --- |
| U6_Forward | ACA GAG AAG ATT AGC ATG GCC |
| U6_Reverse | GAC CAA TTC TCG ATT TGT GCG |
| Unigene_CL8970Contig1_FWD | TCG GCT TCT TCA TTG TGT CC |
| Unigene_CL8970Contig1_REV | AAC CAA CAC TCG GCA TCT TTA |
| Unigene_CL8325_FWD | GAT CCT CCT TGG AGT AAT TGG G |
| Unigene_CL8325_REV | CTG TAG GCA CGT CCT TTC TC |
| Unigene_CL1Contig7021_FWD | CGC ACT ATC TCT TCG TTC ATC A |
| Unigene_CL1Contig7021_REV | CGC TCA GAC AAG GTT AGA GAA G |
| Unigene_CL42980Contig1_FWD | CTA TGG TGG GCA GAT GGT AAA |
| Unigene_CL42980Contig1_REV | AGT AAG AGA AGC AGG TCA AAG G |
| Unigene_Scaffold8176_FWD | GGC ATT GCG ACA AGC ATA AG |
| Unigene_Scaffold8176_REV | GAC CAG GAG CAC AAC AGA TAG |
| Unigene_C95248_FWD | TGC CTC GTC ATA GAA AGG ATT C |
| Unigene_C95248_REV | CCG TTT ACA TGG CTA GTA CTC C |
| Unigene_CL1Contig2785_FWD | CAT TCG ATC CAA AGC TCC ATT TC |
| Unigene_CL1Contig2785_REV | GCC TGA TTA GAG AGT GTC GTA TG |
| Unigene_C133744_FWD | GGC TTG TTT GCA GCT TGT T |
| Unigene_C133744_REV | CGG AAG AGA GAG AGG AGA AGA T |
| Unigene_C71750_FWD | CCT TAC AAC AAC CAG TGA GGT |
| Unigene_C71750_REV | AGG TGG AGA GTT CAA AGA ATC G |
| Unigene_CL1Contig5779_FWD | AGG AGG GCA ACA GAG TCG T |
| Unigene_CL1Contig5779_REV | GAG GCG GAT GAT TCA AGA AA |
| Unigene_CL1Contig328_FWD | GGT ATC CTG GGG AAG CTG AT |
| Unigene_CL1Contig328_REV | GCA CCT ATT CGA AAG CCA AG |
| Unigene_C78790_FWD | ATG GGC TCC TCC AAA AGA TT |
| Unigene_C78790_REV | GGA ATA GAG GGA ACA GCC AAC |
| Unigene_CL8842Contig1_FWD | CAC GGG CAA ATC CAG TAG TT |
| Unigene_CL8842Contig1_REV | CCT CTG ATG GAC TGG ATG CT |
| Unigene_C137988_FWD | GAGGCGCAAACAAAGTCTTC |
| Unigene_C137988_REV | ACTTTTCCACGGAGAGACGA |

**References for Table H**

1. Rigo G, Ayaydin F, Tietz O, Zsigmond L, Kovacs H, Pay A, et al. (2013) Inactivation of plasma membrane-localized CDPK-RELATED KINASE5 decelerates PIN2 exocytosis and root gravitropic response in Arabidopsis. Plant Cell 25: 1592-1608.

2. Xu M, Xie W, Huang M (2012) Overexpression of PeRHD3 alters the root architecture in Populus. Biochem Biophys Res Commun 424: 239-244.

3. Tian H, Jia Y, Niu T, Yu Q, Ding Z (2014) The key players of the primary root growth and development also function in lateral roots in Arabidopsis. Plant Cell Rep 33: 745-753.

4. Deng Y, Wang W, Li WQ, Xia C, Liao HZ, Zhang XQ, et al. (2010) MALE GAMETOPHYTE DEFECTIVE 2, encoding a sialyltransferase-like protein, is required for normal pollen germination and pollen tube growth in Arabidopsis. J Integr Plant Biol 52: 829-843.

5. Takashima S, Abe T, Yoshida S, Kawahigashi H, Saito T, Tsuji S, et al. (2006) Analysis of sialyltransferase-like proteins from Oryza sativa. J Biochem 139: 279-287.

6. Xu R, Zhao H, Dinkins RD, Cheng X, Carberry G, Li QQ (2006) The 73 kD subunit of the cleavage and polyadenylation specificity factor (CPSF) complex affects reproductive development in Arabidopsis. Plant Mol Biol 61: 799-815.

7. Bruggeman Q, Garmier M, de Bont L, Soubigou-Taconnat L, Mazubert C, Benhamed M, et al. (2014). The Polyadenylation Factor Subunit CLEAVAGE AND POLYADENYLATION SPECIFICITY FACTOR30: A Key Factor of Programmed Cell Death and a Regulator of Immunity in Arabidopsis. Plant Physiol 165: 732-746.

8. Dong W, Wang M, Xu F, Quan T, Peng K, Xiao L, et al. (2013) Wheat oxophytodienoate reductase gene TaOPR1 confers salinity tolerance via enhancement of abscisic acid signaling and reactive oxygen species scavenging. Plant Physiol 161: 1217-1228.

9. Tang M, Liu X, Deng H, Shen S (2011) Over-expression of JcDREB, a putative AP2/EREBP domain-containing transcription factor gene in woody biodiesel plant Jatropha curcas, enhances salt and freezing tolerance in transgenic Arabidopsis thaliana. Plant Sci 181: 623-631.

10. Zhang JY, Broeckling CD, Blancaflor EB, Sledge MK, Sumner LW, Wang ZY (2005) Overexpression of WXP1, a putative Medicago truncatula AP2 domain-containing transcription factor gene, increases cuticular wax accumulation and enhances drought tolerance in transgenic alfalfa (Medicago sativa). Plant J 42: 689-707.

11. Wang J, Ding B, Guo Y, Li M, Chen S, Huang G, et al. (2014) Overexpression of a wheat phospholipase D gene, TaPLDalpha, enhances tolerance to drought and osmotic stress in Arabidopsis thaliana. Planta 240: 103-115.

12. Hu J, Baker A, Bartel B, Linka N, Mullen RT, Reumann S, et al. (2012) Plant peroxisomes: biogenesis and function. Plant Cell 24: 2279-2303.

13. Wawer I, Bucholc M, Astier J, Anielska-Mazur A, Dahan J, Kulik A, et al. (2010) Regulation of Nicotiana tabacum osmotic stress-activated protein kinase and its cellular partner GAPDH by nitric oxide in response to salinity. Biochem J 429: 73-83.

14. Hoshi M, Ohki Y, Ito K, Tomita T, Iwatsubo T, Ishimaru Y, et al. (2013) Experimental detection of proteolytic activity in a signal peptide peptidase of Arabidopsis thaliana. BMC Biochem 14:16.

15. Biselli C, Urso S, Bernardo L, Tondelli A, Tacconi G, Martino V, et al. (2010) Identification and mapping of the leaf stripe resistance gene Rdg1a in Hordeum spontaneum. Theor Appl Genet 120: 1207-1218.

16. Tameling WI, Elzinga SD, Darmin PS, Vossen JH, Takken FL, Haring MA, et al. (2002) The tomato R gene products I-2 and MI-1 are functional ATP binding proteins with ATPase activity. Plant Cell 14: 2929-2939.

17. Sun XL, Yu QY, Tang LL, Ji W, Bai X, Cai H, et al. (2013) GsSRK, a G-type lectin S-receptor-like serine/threonine protein kinase, is a positive regulator of plant tolerance to salt stress. J Plant Physiol 170: 505-515.

18. Bogamuwa SP, Jang JC (2014) Tandem CCCH Zinc Finger Proteins in Plant Growth, Development, and Stress Response. Plant Cell Physiol 55: 1367-1375.

19. Dizengremel P, Le Thiec D, Hasenfratz-Sauder MP, Vaultier MN, Bagard M, Jolivet Y (2009) Metabolic-dependent changes in plant cell redox power after ozone exposure. Plant Biol (Stuttg) 11 Suppl 1: 35-42.

20. Holtgrefe S, Gohlke J, Starmann J, Druce S, Klocke S, Altmann B, et al. (2008) Regulation of plant cytosolic glyceraldehyde 3-phosphate dehydrogenase isoforms by thiol modifications. Physiol Plant 133: 211-228.

21. Jaillais Y, Fobis-Loisy I, Miege C, Rollin C, Gaude T (2006) AtSNX1 defines an endosome for auxin-carrier trafficking in Arabidopsis. Nature 443: 106-109.

22. Abel S, Burstenbinder K, Muller J (2013) The emerging function of IQD proteins as scaffolds in cellular signaling and trafficking. Plant Signal Behav 8: e24369.

23. Davy A, Thomsen KK, Juliano MA, Alves LC, Svendsen I, Simpson DJ (2000) Purification and characterization of barley dipeptidyl peptidase IV. Plant Physiol 122: 425-432.

24. Mobley EM, Kunkel BN, Keith B (1999) Identification, characterization and comparative analysis of a novel chorismate mutase gene in Arabidopsis thaliana. Gene 240: 115-123.

25. Ouyang B, Yang T, Li H, Zhang L, Zhang Y, Zhang J, et al. (2007) Identification of early salt stress response genes in tomato root by suppression subtractive hybridization and microarray analysis. J Exp Bot 58: 507-520.

26. Chopra S, Palencia A, Virus C, Tripathy A, Temple BR, Velazquez-Campoy A, et al. (2013) Plant tumour biocontrol agent employs a tRNA-dependent mechanism to inhibit leucyl-tRNA synthetase. Nat Commun 4: 1417.

27. Guo W, Zuo Z, Cheng X, Sun J, Li H, Li L, et al. (2014) The chloride channel family gene CLCd negatively regulates pathogen-associated molecular pattern (PAMP)-triggered immunity in Arabidopsis. J Exp Bot 65: 1205-1215.

28. Uniprot (<http://www.uniprot.org/uniprot/Q9FPT5>; accessed: 08-08-2014).

29. Arab A, Alves MN, Sartoratto A, Ogasawara DC, Trigo JR (2012) Methyl jasmonate increases the tropane alkaloid scopolamine and reduces natural herbivory in brugmansia suaveolens: is scopolamine responsible for plant resistance? Neotrop Entomol 41: 2-8.

30. Rowland O, Ludwig AA, Merrick CJ, Baillieul F, Tracy FE, Durrant WE, et al. (2005) Functional analysis of Avr9/Cf-9 rapidly elicited genes identifies a protein kinase, ACIK1, that is essential for full Cf-9-dependent disease resistance in tomato. Plant Cell 17: 295-310.

31. van den Burg HA, Tsitsigiannis DI, Rowland O, Lo J, Rallapalli G, Maclean D, et al. (2008) The F-box protein ACRE189/ACIF1 regulates cell death and defense responses activated during pathogen recognition in tobacco and tomato. Plant Cell 20: 697-719.

32. Liang M, Haroldsen V, Cai X, Wu Y (2006) Expression of a putative laccase gene, ZmLAC1, in maize primary roots under stress. Plant Cell Environ 29:746-753.

33. Wei J, Tirajoh A, Effendy J, Plant AL (2000) Characterization of salt-induced changes in gene expression in tomato (Lycopersicon esculentum) roots and the role played by abscisic acid. Plant Sci 159: 135-148.

34. Tran LT, Taylor JS, Constabel CP (2012) The polyphenol oxidase gene family in land plants: Lineage-specific duplication and expansion. BMC Genomics 13: 395.

35. Yang Y, Sun X, Yang S, Li X (2014) Molecular cloning and characterization of a novel SK3-type dehydrin gene from Stipa purpurea. Biochem Biophys Res Commun 448: 145-150.

36. Turan S, Cornish K, Kumar S (2012) Salinity tolerance in plants: Breeding and genetic engineering. Aust J Crop Sci 6: 1337-1348.
